# Multiscale Insights into the Genesis of Pickering Emulsions: Nanomixing and Interfacial Design of Surface-Active Silica Particles

**DOI:** 10.1021/acs.langmuir.5c05703

**Published:** 2026-03-04

**Authors:** Kang Wang, Antoni Salom-Català, Alberto Roldan, Marc Pera-Titus

**Affiliations:** Cardiff Catalysis Institute, Translational Research Hub, 2112 Cardiff University, Maindy Road, Cardiff CF24 4HQ, United Kingdom

## Abstract

Pickering emulsions—liquid–liquid dispersions stabilized by solid particles—offer a sustainable route for oil extraction, fine chemistry, organic synthesis, and catalysis applications. The formulation of Pickering emulsions involves surface-active particles that selectively adsorb at the interface between immiscible liquids. However, the nanoscale mechanisms that govern particle adsorption, interfacial nanostructuring, and emulsion stability remain elusive. Here, we combined molecular dynamics and dissipative particle dynamics simulations with emulsification experiments to elucidate how the length, surface density, and architecture (Janus vs homogeneous) of aliphatic ligands grafted on silica particles dictate interfacial assembly and emulsion formation in the toluene–water system. We found that longer aliphatic chains enhance the interfacial organization and stability at lower surface coverage, improving both cost efficiency and sustainability. Moreover, Janus architectures generated more robust emulsions and exhibited distinct interfacial nanomixing behavior compared with homogeneous particles. The simulations accurately predicted phase inversion transitions, consistent with experimental observations coupling water–toluene nanomixing at the microscale to emulsification at the mesoscale. This combined computational–experimental approach revealed new design rules for engineering particle–liquid interfaces with tailored stability and functionality at the nanoscale, with potential impact for surface-active catalyst design.

## Graphical abstract

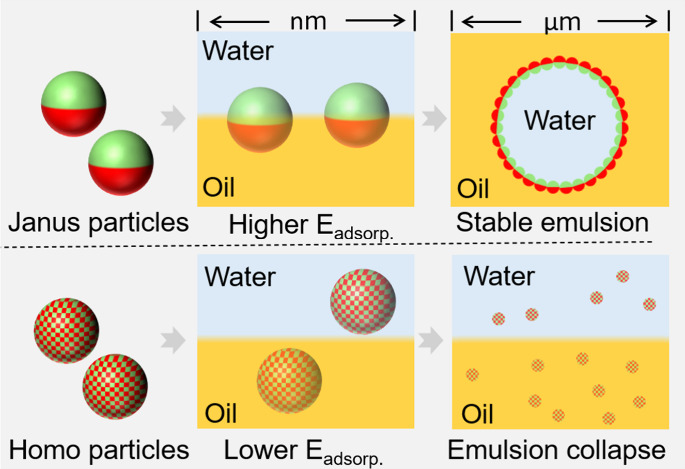

## Introduction

Pickering emulsions—liquid–liquid dispersions stabilized by solid particles rather than molecular surfactants—have attracted significant attention as sustainable alternatives for stabilizing multiphase systems.^1^ Since the pioneering work of Ramsden and Pickering in the early 20th century,^2,3^ particle-stabilized emulsions have evolved from a colloidal curiosity into versatile platforms for applications ranging from catalysis and drug delivery to food processing and energy materials. In such systems, surface-active particles adsorb irreversibly at the oil–water interface, lowering the interfacial energy and forming a physical barrier that suppresses droplet coalescence.^4–6^ Unlike conventional surfactants, solid particles can impart long-term stability, environmental compatibility, and tunable interfacial functionality, making them ideal for designing responsive, recyclable, and sustainable emulsions.

Despite this extensive empirical progress, the fundamental nanoscale mechanisms governing particle adsorption, self-assembly, and interfacial structuring remain elusive. The interfacial behavior of colloidal particles depends sensitively on their size, shape, surface chemistry, and wettability, all of which condition the nature and strength of intermolecular interactions and determine the particle’s affinity for each fluid phase.^7–9^ Among these parameters, surface functionalization with organic ligands plays a decisive role: the nature, length, density, and spatial distribution of grafted molecules regulate the interfacial energy balance and dynamic rearrangement at the liquid–liquid boundary. However, the relationship between the molecular-level ligand architecture and macroscopic emulsion stability remains poorly quantified, largely due to the complex, multiscale dynamics spanning nanometers to micrometers and nanoseconds to seconds.

Recent advances in molecular and mesoscale simulations now enable a detailed examination of these interfacial processes. Molecular dynamics (MD) captures the atomistic behavior of grafted ligands, solvent penetration, and nanoscale interfacial structuring,^10–12^ while dissipative particle dynamics (DPD) extends the accessible time and length scales to capture droplet formation and stabilization^13–15^ and model distribution of liquids on particles.^16–20^ The combination of these complementary approaches, supported by targeted experiments, provides a powerful framework for developing predictive models that bridge the molecular design and macroscopic performance.

In this context, Janus particles—asymmetric colloids with two chemically distinct hemispheres—represent a particularly promising class of interfacial stabilizers. Their amphiphilic character allows selective orientation at oil–water interfaces, enhancing adsorption strength and interfacial packing with up to a 3-fold increase in detachment energy compared to randomly functionalized counterparts.^21–25^ Janus particles can generate emulsions, where the interfacial particle self-assembly, orientation, and droplet morphology can be governed by the type and density of functional groups on each hemisphere.^26^ Nonetheless, quantitative understanding of how Janus architecture interacts with other design variables—such as ligand chain length and surface coverage—to influence emulsion stability and phase behavior is still lacking.

Herein, we integrate molecular dynamics and dissipative particle dynamics simulations with systematic emulsification experiments to elucidate how the length (C3, C9, and C18), surface density, and spatial arrangement (homogeneous vs Janus) of aliphatic ligands grafted on silica particles dictate their interfacial assembly and emulsion stability in the toluene–water system. This multiscale approach enables direct correlation between nanoscale ligand organization, interfacial nanomixing, and macroscopic emulsion behavior, offering molecular-level insight into the genesis of Pickering emulsions. We demonstrate that longer aliphatic chains enhance stability at lower grafting densities, while Janus architectures yield superior interfacial adsorption and broader stability windows. These findings establish design principles for engineering particle–liquid interfaces with tunable properties, advancing the rational design of emulsifiers for catalytic, separation, and energy applications.

## Simulation Details

### Models and Interaction Parameters

#### Dissipative Particle Dynamics (DPD)

DPD is based on a coarse-graining approach in which a group of atoms of the real system is embedded into a “bead”, allowing the simulation of a mesoscopic system.^27–29^ The system considered in this study consisted of two immiscible liquids, toluene (T) and water (W), and a set of functionalized organosilica particles. The hydrophilic ligands grafted onto the particle surface were hydroxyl groups (OH), whereas the hydrophobic ligands were based on hydrocarbon chains (CH). Each OH group was represented by a single bead, and the hydrophobic beads corresponded to a propane unit. Figure 1 depicts the coarse-grained models for all components of the real system.

**Figure 1:**
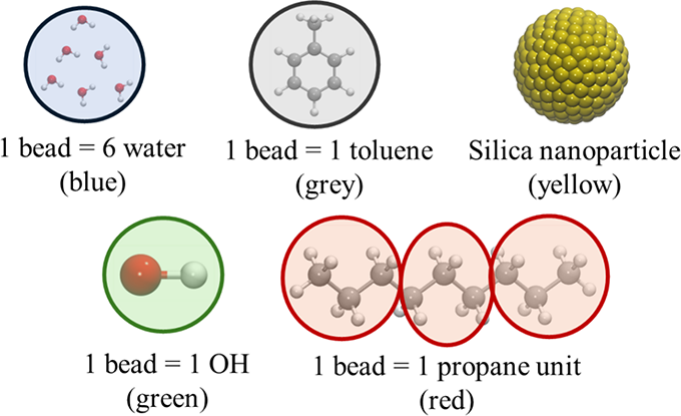
Schematic representation of the coarse-grained system. The colors in parentheses represent the color of the beads.

The bead volume was set to 180 Å^3^, corresponding to a water/toluene molar ratio of 6.0, where each water bead corresponded to six water molecules. Each toluene bead simulated one toluene molecule. The number density of the system, ρ*r*_c_^3^, defined as the number of beads in a cube of side *r*_c_, was set to 3, leading to the distance unit of *r*_c_ = (3 × 180)^1/3^ = 8.14 Å. With this value, the mass densities of the water and toluene phases were 0.997 and 0.85 g/cm^3^, respectively, in agreement with the experimental values at 298 K.^30^

For simplicity, the particle core was modeled as a rigid sphere composed of a central bead, an inner shell, and an outer sphere representing the surface.^31^ To accelerate the calculations, the radii of all particles were set at 1 *r*_c_. The propane beads within the hydrocarbon chains were linked with a harmonic spring with a force constant of *k* = 100 and an equilibrium distance of *r*_0_ = 0.7*r*_c_.^32,33^ The predicted T/W interfacial tension was 36.04 mN/m, comparable with the experimental value (37.6 mN/m).^34^

We conducted DPD calculations for three different chain lengths: 1 bead (C_3_H_7_), 3 beads (C_9_H_19_), and 6 beads (C_18_H_37_), labeled as **TB1**, **TB3**, and **TB6**, respectively. Additionally, we considered two different surface architectures: Janus and homogeneous distributions of the alkyl chains. For Janus architectures, the hydrophobic ligands (alkyl) were placed on one side of the particle while the hydrophilic ligands (−OH) were placed on the other side. We performed one calculation for each ligand density for the three different chain lengths. For homogeneous architectures, we calculated three different dispositions of the ligands: one where the ligands were homogeneously distributed and the other two where the ligands were randomly distributed on the surface. Desorption energies were measured by averaging the three punctual energies. For the sake of simplicity, the model was simplified to have one ligand for each silica bead on the surface, corresponding to monopodal grafting. We set the total SiOH density at 26 groups/nm^2^ for **TB1** chains, whereas it was 24 groups/nm^2^ for **TB3** and **TB6** chains. These values are comparable to the total SiOH density of the pristine silica particles, i.e., ∼70 groups/nm^2^, as inferred from combined thermogravimetric analysis (TGA) and BET specific surface areas.

Since the rigid spheres used to simulate the particle core and shell were predefined to ensure the best bead packing,^35^ we selected spheres of 212 and 200 points to represent the particle surface with **TB1** and **TB3**/**TB6** chains, respectively, taking into account the particle’s radius of 1 *r*_c_, leading to an area of 8.33 nm^2^. The **TB1** particle core was formed by 425 silica beads, whereas the **TB3** and **TB6** particle cores were composed of 401 silica beads each. The first ligand shell was formed by the corresponding number of hydrophilic and silica beads on which the hydrophobic beads were bonded. The reason for these extra silica beads on the particle surface was to keep the length difference between the Si–OH and propane groups. Figure 2 shows an example of Janus and homogeneous particles used in this work. The DPD interaction parameters for all components used in the simulations, the parametrization method, and computation details are compiled in the Supporting Information (Figures S1 and S2 and Table S1, see Sections S1.1–1.3).

**Figure 2:**
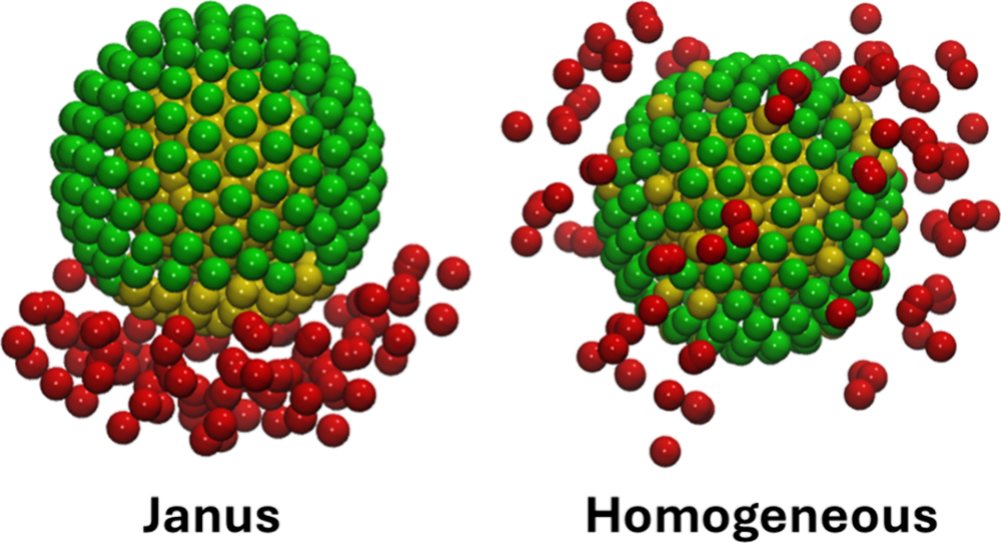
Examples of Janus (left) and homogeneous (right) particles with **TB3** chains used in the simulations. The surface density of propane chains was 4.02 groups/nm^2^. Yellow, green, and red beads represent silica, OH, and propane units, respectively.

### Energies of Interfacial Particle Adsorption and Emulsion Formation

The free energy of adsorption of a single, spherical particle at the O/W interface from one liquid phase, attributable to changes in interface areas and contact lines, Δ_int_ *G*_p_, can be expressed using eq 1 earlier developed by Aveyard and Clint^36–38^ and further confirmed by Kruglyakov and Nushtayeva,^39^ by comparison of the free energy of a particle adsorbed at the L-L interface and the free energy of a particle dispersed in one of the liquid phases (see derivation details in ref 40):

$$\Delta_{\mathrm{int}}G_{p}=-\frac{\pi }{4}d_{p}^{2}\gamma_{\mathrm{OW}}\left(d_{p}/D_{D}\right)\left[{\left(1\pm \cos\;\theta \right)}^{2}-\frac{4\tau_{\mathrm{OW}}}{d_{p}\gamma_{\mathrm{OW}}\;\sin\;\theta }\left(1\pm \cos\;\theta \right)\right]$$ 1

where θ is the 3-phase interfacial contact angle (measured through the water phase, see definition in Figure S3), *d*_p_ is the average particle diameter, γ_OW_ is the effective O/W interfacial tension function of the O/W interface curvature that depends on the *d*_p_/*D*_D_ ratio (*D*_D_ = swollen droplet diameter),^41^ and τ_OW_ is the line tension. The (+) and (−) signs within the brackets correspond to particle removal into the bulk oil and water phases, respectively.

Equation 1 can be corrected with terms including electrostatic and van der Waals interactions between the adsorbing particles and the particle film. These interactions are often repulsive and can contribute by 20–300 kT to Δ_int_ *G*_p_ at high particle coverages (0.97–0.99) for contact angles in the range of 50–150°.^42^

If *d*_p_/*D*_D_ ≪0.1 and τ ≪ *r*γ_OW_, eq 1 can be simplified to the well-known expression that is commonly reported in reviews and manuals:

$$\Delta_{\mathrm{int}}G_{p}=-\frac{\pi }{4}d_{p}^{2}\gamma_{\mathrm{OW}}{\left(1\pm \cos\;\theta \right)}^{2}$$ 2

As inferred from eqs 1 and 2, the optimal contact angle for single particle adsorption is 90°, as this angle corresponds to the point where the particle’s desorption energy peaks. Positive line tensions reduce the length of the contact line and push the contact angle far from 90°, whereas negative line tensions shift the contact angle toward 90°. Equation 2 can be expressed in a dimensionless form as follows:

$$E_{p,\dim}=-\frac{4}{\pi }\frac{\Delta_{\mathrm{int}}G_{p}}{d_{p}^{2}\gamma_{\mathrm{OW}}}={\left(1\pm \cos\;\theta \right)}^{2}$$ 3

The plot of *E*_p,dim_ against the surface density for the different particles allows the demarcation of stability zones for oil−water emulsification and the determination of the emulsion type. In our approach, we considered the range of 0.23–0.85 for *E*_p,dim_, corresponding to the θ_OW,C_ ranges of 60–85° (O/W emulsions) and 95–120° (O/W emulsions), ensuring emulsion stability.

The free energy of particle-coated droplet formation, Δ*G*_droplet_, can be computed using the expression earlier proposed by Kralchevsky and co-workers (eq 4) by assuming that droplets behave as hard spheres,^43^ and where *n*_p_ is the number of particles adsorbed at the water–toluene interface.

$$\Delta G_{\mathrm{droplet}}=\left(4\pi D_{D}^{2}\gamma_{\mathrm{OW}}+n_{p}\Delta_{\mathrm{int}}G_{p}\right)$$ 4

#### Classical Molecular Dynamics (MD)

To investigate the distribution of water and toluene on the particle surface, we performed a series of classical MD simulations. The system consisted of a silica slab based on an α-cristobalite motif, following the model proposed by Emami et al.^44^ The structure was used as a template to graft the alkyl chains and OH ligands involved in the nanomixing process on the studied nanoparticles. The surface density of the silanol groups was set to 4.7 groups/nm^2^, with only one OH group grafted onto each surface silicon atom. The surface was modified to simulate the hydrophobic part by grafting silane groups on selected positions. Specifically, for each set of three neigboring OH ligands, the hydrogen atoms were removedand the three O atoms were bonded to a silicon atom in a tripodal configuration. Two different alkyl chain lenghts were considered: a propyl chain (C_3_) and a nonyl chain (C_9_). For both chain lengths, silane groups were arranged in two distinct spatial configurations:conformations, i.e., Janus and homogeneous. Furthermore, two different initial configurations of toluene and water molecules were examines, i.e., completely mixed or completely separated (Figure 3 and Figure S4). In both cases, Regardless of the initial conditions (i.e., a 1:1 T/W mixture or 1:1 fully separated phases), the same equilibrium states were achieved. For the Janus surface, the silane and OH group densities were 1.03 and 1.72 groups/nm^2^, respectively. For the homogeneous surface, the corresponding densities were 0.86 and 2.24 groups/nm^2^, respectively. To avoid artifical asymmetry arising from periodic boundary conditions, silane groups were placed on both the top and bottom surfaces of the slab in identical spatial distribution, thereby ensuring equivalent interactions with water and toluene molecules on both sides of the slab.

**Figure 3:**
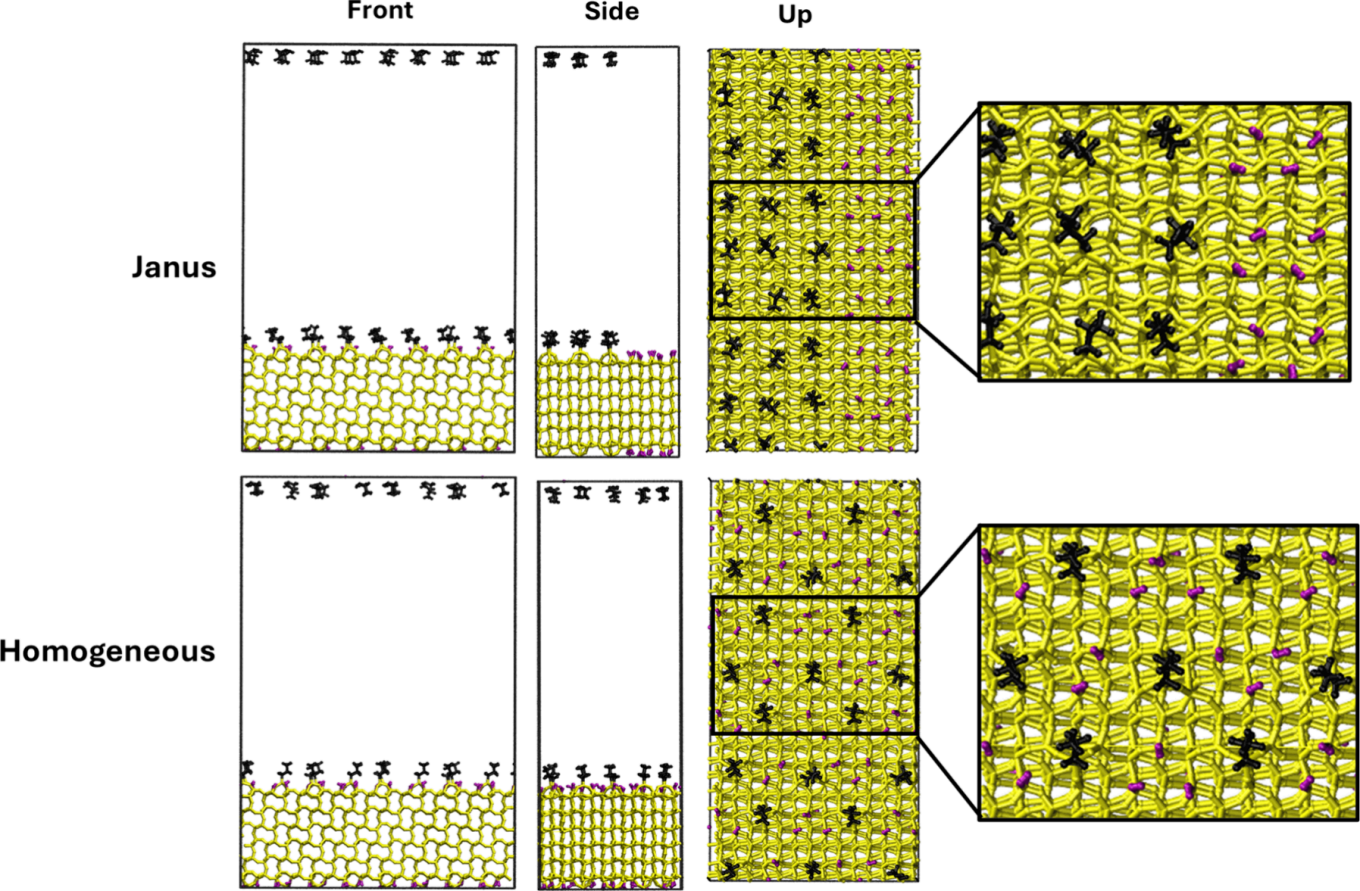
Slab models were used in this work for the MD calculations. The C_9_ chain length slabs were built in the same manner but with longer aliphatic chains. The C and H atoms of the chains are in black, and the O and H of the OH groups are in purple.

## Results and Discussion

### Preparation and Characterization of Surface-Active Silica Particles

Monodisperse silica particles (∼250 nm) were synthesized via the Stöber method using tetraethyl orthosilicate (TEOS) as the silica precursor, ammonia as the catalyst, and ethanol as the solvent (see the Supporting Information for details).^45^ Janus particles (JPs) were obtained by the Pickering emulsion template method, selectively functionalizing one hemisphere of the pristine silica with alkylsilanes of varying chain lengths (propyl, C3; octyl, C8). Homogeneously functionalized silica particles (HPs) were prepared by grafting the same silanes uniformly across the surface without the paraffin wax. The resulting particle series—C3-JP-10, C3-JP-20, C8-JP-10, C8-JP-20, C3-HP-10, C3-HP-20, C8-HP-10, and C8-HP-20—span variations in both the alkyl chain length and silane precursor volume (10 or 20 μL).

Thermogravimetric analysis (TGA) revealed three distinct weight loss regions corresponding to adsorbed water desorption (30–150 °C), combustion of organic chains (150–450 °C), and condensation of residual silanol groups (450–900 °C) (Figure S5).^46^ Table S2 lists the weight loss values for the different particles together with the surface density of propyl/octyl chains and SiOH groups. All of the samples display a similar weight loss for the first region (range 0.32–0.91%). Both Janus and homogeneous particles exhibit comparable weight mass losses in the range of 150–450 °C for propyl and octyl chains, indicating similar grafting densities for identical silane dosages. As expected, reducing the silane input (from 20 to 10 μL) leads to lower surface coverage.

Table 1 summarizes the key physicochemical parameters for the different particles. For C3-functionalized samples, the grafting density decreases from 27.0 to 24.6 groups nm^–2^ with reduced silane addition, while C8-functionalized particles display lower overall coverage (11.1–10.3 groups nm^–2^). The hydrophilic–lipophilic balance (HLB) values range from 1.59 to 5.80, consistent with increasingly hydrophobic surfaces at higher alkylation levels. TEM and BET analyses (Table 1 and Figure S6) confirm that silane grafting does not alter the particle morphology or surface area, underscoring that chemical modification occurs primarily at the external surface without affecting particle size or porosity, consistent with previous findings on Janus and homogeneous particles.^47^

**Table 1:** Textural and Surface Properties of Surface-Active Silica Particles (253 ± 5 nm) Prepared in This Study^g^

variable	C3-JP-20	C3-JP-10	C3-HP-20	C3-HP-10	C8-JP-20	C8-JP-10	C8-HP-20	C8-HP-10
*C_n_* (groups/nm^2^)^a^	27.0	24.6	27.0	24.6	11.1	10.3	11.1	10.3
SiOH (groups/nm^2^)^a^	43.0	45.4	43.0	45.4	58.9	59.7	58.9	59.7
HLB^b^	1.59	1.84	1.59	1.84	5.31	5.80	5.31	5.80
Φ (−)^c^	1.02	0.92	1.29	1.17	0.54	0.64	0.48	0.43
Γ (SiNP/μm^2^)	21	18	27	25	20	19	27	24
θ_TW_ (°)^d^	81	85	104	105	133	125	143	143
emulsion type^e^	W/O	O/W	W/O	O/W	W/O	O/W	W/O	O/W
droplet size (μm)^f^	251	224	331	302	242	231	322	289

a Measured by TGA.

b HLB (hydrophilic–lipophilic balance of particles) is calculated by hydrophilic group number/lipophilic group number.

c Measured from the plot of 1/®D vs SiNP loading in the range 0.5–1.5 wt % (Figure 5).

d Measured from DPD simulations.

e Measured by the dilution test.

f Measured emulsions containing 0.5 wt % of SiNPs with a 1:1 toluene/water volume ratio.

g Listed are grafting densities of alkyl chains (*C_n_*) and surface silanol groups (SiOH), hydrophilic–lipophilic balance (HLB), surface coverage (Φ), interfacial particle density (Γ), water contact angle at the toluene–water interface (θ_TW_), emulsion type, and average droplet size. The data highlight the influence of alkyl chain length, surface density, and particle architecture (Janus vs homogeneous) on interfacial wettability and emulsion stability.

### Emulsification Behavior in the Toluene/Water System

The emulsification performance of Janus and homogeneous particles was examined using the toluene/water (1:1 v/v) system under identical homogenization conditions (30,000 rpm, 15 s) (see details in the Supporting Information, Section S2.5). The resulting emulsion morphology and stability were markedly influenced by both the particle architecture and surface chemistry (Figure 4).

**Figure 4:**
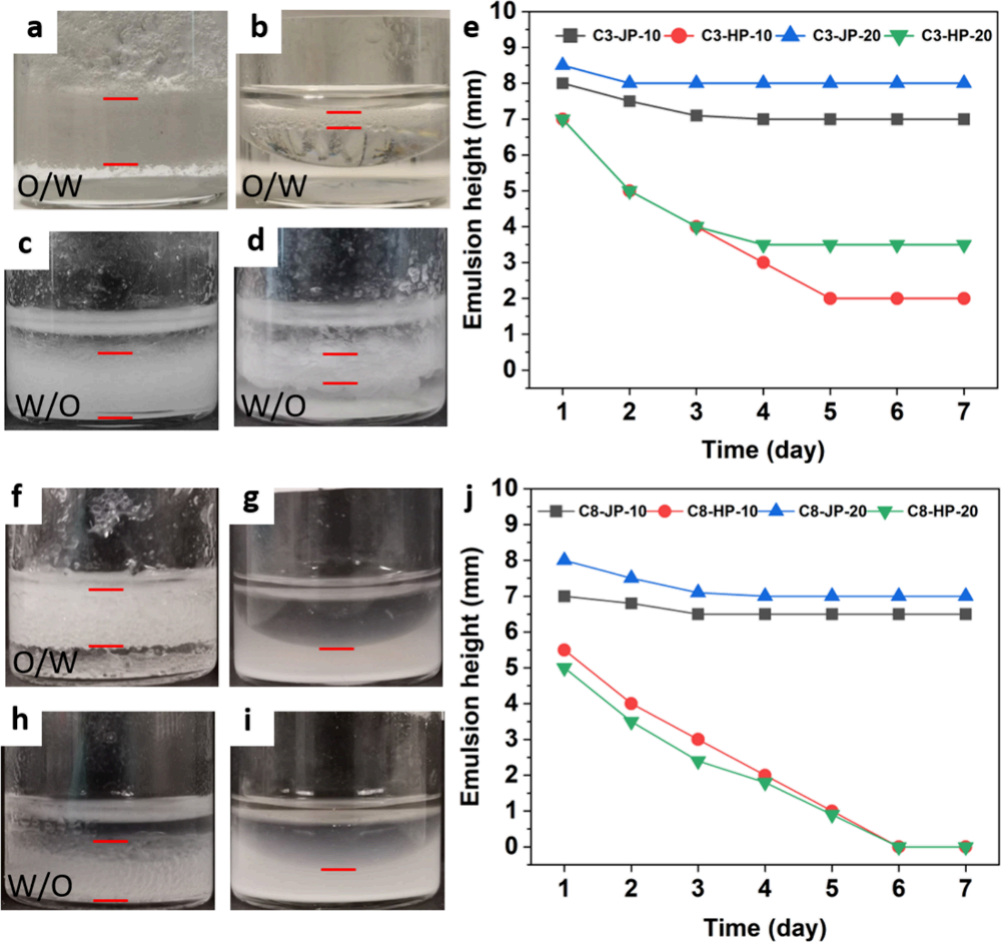
Emulsification behavior of C3- and C8-functionalized silica particles in the toluene/water (1:1 v/v) system. Optical images of emulsions obtained after 7 h under static conditions stabilized by (a) C3-JP-10, (b) C3-HP-10, (c) C3-JP-20, (d) C3-HP-20, (f) C8-JP-10, (g) C8-HP-10, (h) C8-JP-20, and (i) C8-HP-20 after 7 days under static conditions. (e, j) Temporal evolution of emulsion height for C3- and C8-grafted systems. Janus particles consistently yield higher stability and longer emulsion lifetimes than homogeneous analogues, irrespective of the grafting density or chain length. Emulsification conditions: 9.3 mg of particles, 1 mL of toluene, 1 mL of water, homogenization at 30,000 rpm for 15 s.

Particles grafted with short alkyl chains (C3) generate either O/W or W/O emulsions depending on the grafting density. C3-JP-10 and C3-HP-10 both yield O/W emulsions, but the Janus architecture affords significantly higher stability—emulsion heights of 7.0 and 2.0 mm after 7 days under static conditions, respectively (Figure 4a,b). Increasing the grafting density (C3-JP-20 and C3-HP-20) inverts the emulsion type to W/O, but the Janus particles again produce taller and more stable emulsions with heights of 8.0 and 3.5 mm, respectively, after 7 days (Figure 4c,d). Similar trends are observed for C8-functionalized particles: C8-JP-10 generates stable O/W emulsions, while C8-JP-20 yields W/O emulsions with a height around 6.5 mm (Figure 4f,h). In contrast, none of the C8-HP particles stabilize persistent emulsions and disperse in the water phase (Figure 4g,i). Remarkably, C3-JP-10 emulsions remain unchanged after six months under ambient conditions, underscoring their exceptional long-term stability.

The time evolution of the emulsion height (Figure 4e,j) exhibits minimal decay for all Janus-based emulsions, whereas those stabilized by homogeneous particles collapse over a 7-day period. The emulsion heights for C3-HP-10 and C3-HP-20 decrease significantly from 7 to 2 and 3.5 mm, respectively, within 5 and 4 days, and remain stable thereafter. C8-HP-10 and C8-HP-20 show much lower stability, with the emulsion height vanishing completely from the initial 5 mm after 6 days. Droplet size distributions (Table 1 and Figure S7) corroborate these observations: emulsions stabilized by Janus particles display smaller, more uniform droplets (224–289 μm) compared with their homogeneous counterparts (302–331 μm). For all particles, a lower surface density of chains results in slightly smaller droplets. Increasing particle loading further reduces droplet size, following an inverse scaling relationship (Figure 5). This behavior reflects efficient and homogeneous particle adsorption at the oil–water interface, indicative of strong interfacial anchoring and suppressed coalescence.

**Figure 5:**
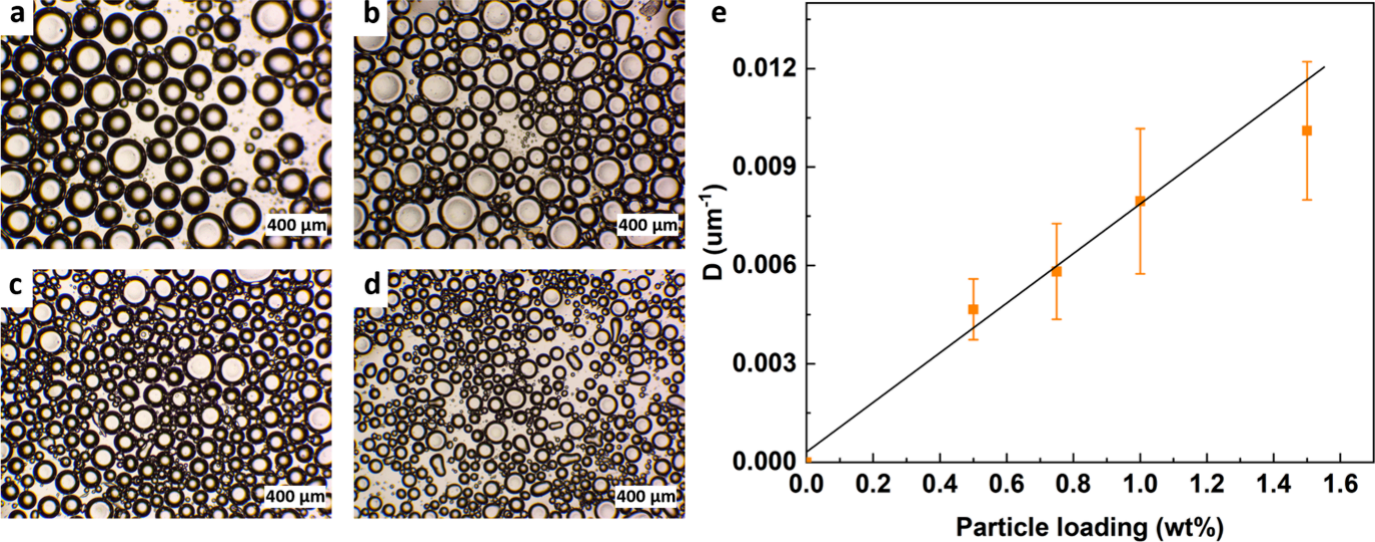
Effect of particle loading on emulsion droplet size for C3-JP-10 particles. (a–d) Optical micrographs of emulsions prepared with particle loadings of 0.5, 0.75, 1.0, and 1.5 wt %. (e) Inverse average droplet diameter (1/*D*) as a function of SiNP concentration. The linear dependence indicates homogeneous adsorption and the efficient self-assembly of Janus particles at the oil–water interface. Emulsification conditions: toluene/water (1:1 v/v), homogenization at 30,000 rpm for 15 s, 25 °C.

Interfacial coverage calculations (Table 1) reveal that C3-functionalized Janus particles form nearly complete monolayers (Φ = 0.92–1.02 and 18–21 particles/μm^2^), while homogeneous analogues exhibited slightly denser packing (Φ = 1.17–1.29, Γ = 25–27 particles/μm^2^) (see Section S2.6 in the SI for details). In contrast, C8-grafted particles exhibit submonolayer particle adsorption with a surface coverage slightly higher for Janus particles compared to homogeneous particles (Φ = 0.54–0.64 vs 0.43–0.48), whereas the surface density of particles is lower for Janus particles compared to homogeneous particles (19–20 vs 24–27 particles/μm^2^). Despite their higher surface density, the homogeneous particles lead to less stable emulsions. This counterintuitive behavior can be explained by the much lower contact angles for C8-grafted homogeneous particles compared to Janus particles, resulting in a much lower detachment energy.

### Interfacial Adsorption Energetics

To rationalize the experimental trends, we estimated the contact angles of three different simulated particles grafted with C3, C9, and C18 chains via DPD simulations (see computational details), and from these values, we computed the *E*_dim_ energies for all particles (Figure 6). The *E*_dim_ values vary systematically with the HLB and surface chain density. Janus particles exhibit broad adsorption windows with maximum stability at intermediate HLB values (3–5), while homogeneous particles adsorb at higher HLBs (5–45) and display narrower stability ranges. For both particle architectures, increasing the alkyl chain length from C3 to C9 enhances the interfacial anchoring, but a further extension to C18 produces minimal gains, indicating a saturation effect. Notably, while homogeneous particles show slightly lower optimal surface densities, their narrow stability window renders them less effective stabilizers in practice. The simulations predict that low chain densities (high HLB) favor the emulsion of W/O, whereas dense hydrophobic coverage (low HLB) promotes W/O emulsions, in agreement with experimental observations.

**Figure 6:**
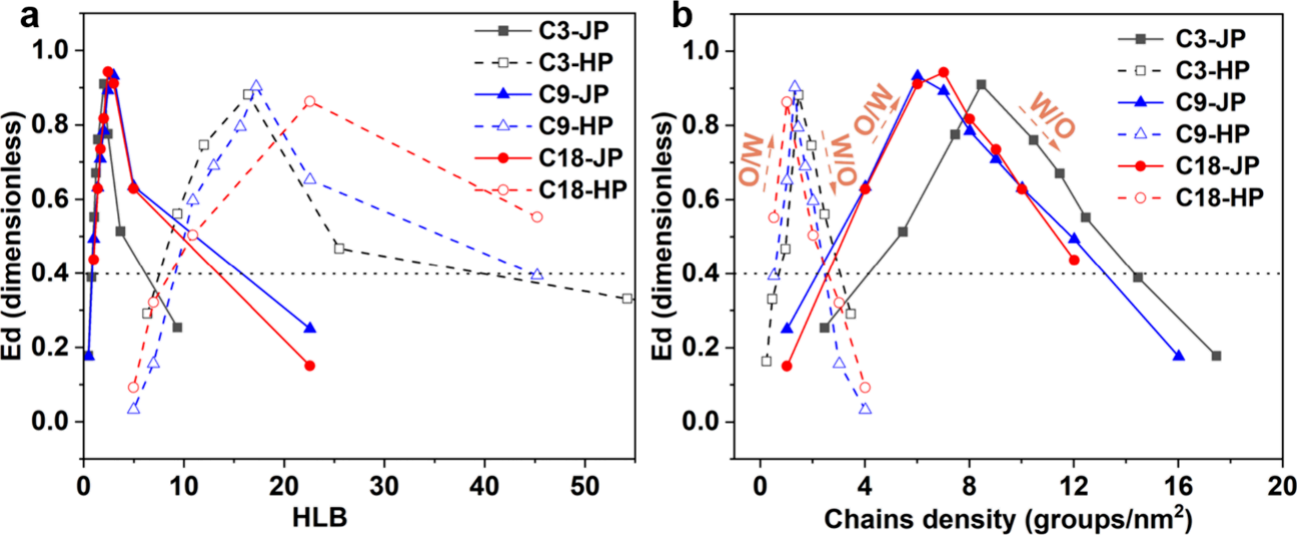
Simulated interfacial adsorption energies (*E*_dim_) as a function of particle hydrophilic–lipophilic balance (HLB) and alkyl chain density. (a) *E*_dim_ vs HLB and (b) *E*_dim_ vs chain density for Janus (solid curves) and homogeneous (dashed curves) particles grafted with C3, C9, and C18 chains. Janus particles exhibit broader stability windows and higher maximum adsorption energies, indicating stronger interfacial anchoring. The dashed black line (*E*_dim_ ≈ 0.4) marks the lower stability limit.

Free energy analyses of droplet formation (Figure 7 and Table S3) further reveal that emulsions stabilized by Janus particles possess lower (less positive, i.e., more favorable) interfacial free energies of droplet formation than those stabilized by homogeneous analogues. This difference accounts for the enhanced emulsion stability and smaller droplet sizes observed experimentally.

**Figure 7:**
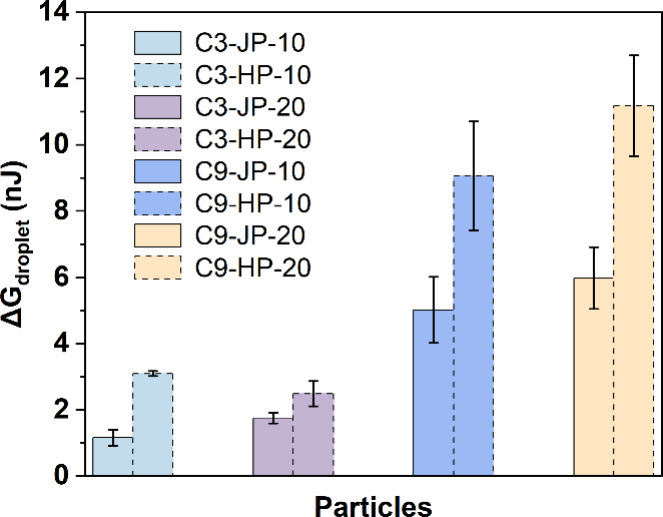
Free energy of droplet formation (Δ*G*_droplet_) for emulsions stabilized by Janus and homogeneous particles. Janus particles functionalized with C3 and C9 chains display significantly lower Δ*G*_droplet_ values, reflecting more favorable interfacial stabilization compared to their homogeneous counterparts. These results rationalize the experimentally observed superior emulsion stability and smaller droplet sizes for Janus-stabilized systems.

### Local Toluene–Water Miscibility on Particle Surfaces

Classical molecular dynamics (MD) simulations were employed to probe nanoscale solvent organization around the grafted particles. C3- and C9-functionalized Janus and homogeneous particles were modeled at equivalent grafting densities (≈1.0 chains nm^–2^).

For C3-grafted particles (Figure 8a1,b1), water molecules predominantly wet the surface, while toluene molecules remain largely excluded and locate predominantly in the center of the simulation box, although occasional penetration events indicate partial local miscibility. In Janus particles, this nanomixing occurs exclusively on the hydrophobic hemisphere.

**Figure 8:**
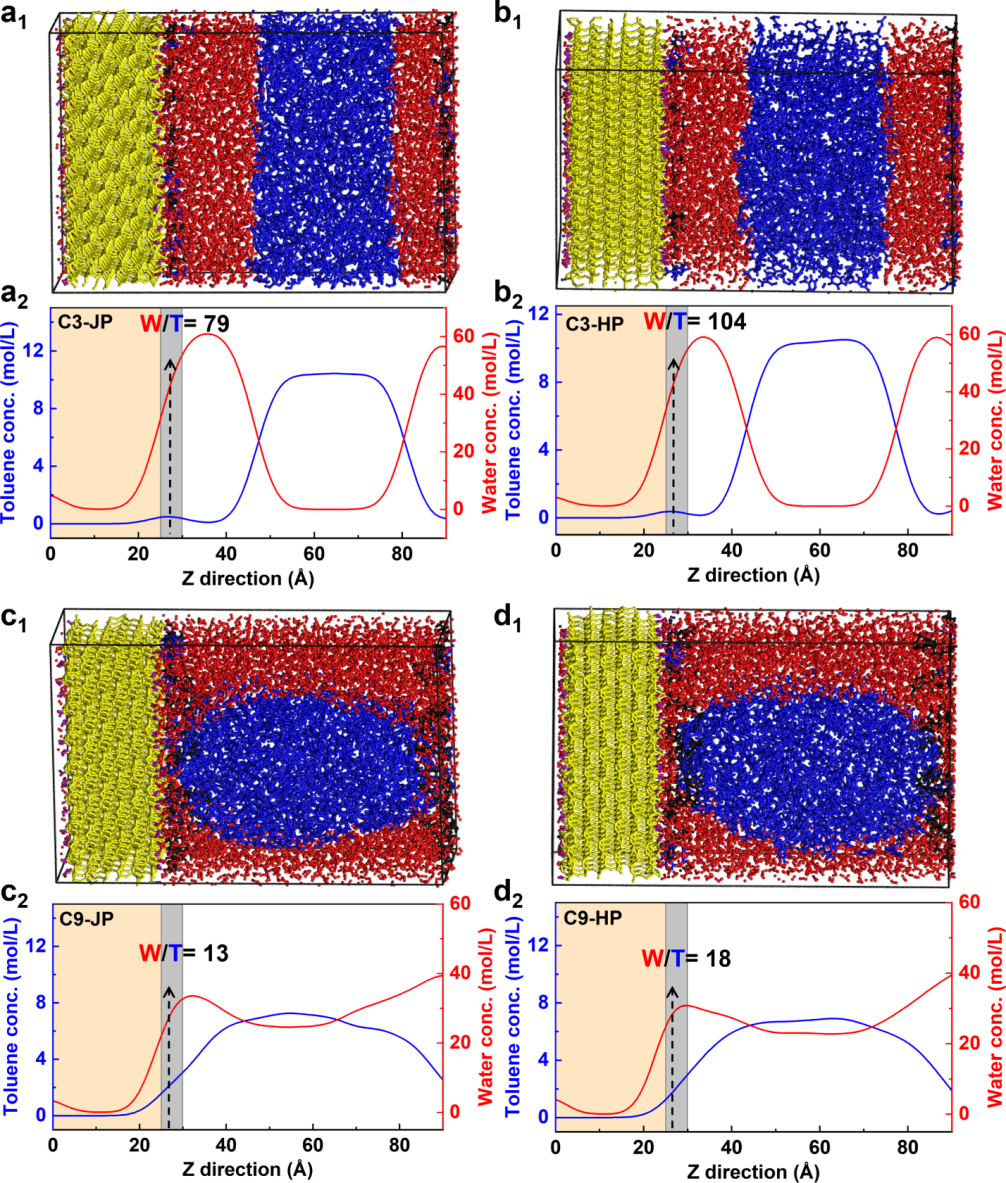
Molecular dynamics (MD) simulation snapshots and local solvent concentration profiles near C3- and C9-grafted silica particles. Final simulation frames (50 ns) for (a1) C3-JP, (b1) C3-HP, (c1) C9-JP, and (d1) C9-HP, showing solvent organization around the grafted surfaces. Yellow = Si/O atoms of the substrate; black = C/H of alkyl chains; purple = hydrophilic groups; blue = toluene; red = water. (a2–d2) Corresponding local concentration profiles averaged along the *z*-axis, indicating distinct nanomixing behavior: (a_2_) C3-JP, (b_2_) C3-HP, (c_2_) C9-JP, and (d_2_) C9-HP. The shaded gray region marks the interfacial zone. The number in the small plot is the local concentration of toluene in mol/L at the interface (26 Å). Red = water, blue = toluene. Janus particles, particularly C9-JP, promote pronounced local toluene–water mixing on the hydrophobic hemisphere, correlating with enhanced emulsion stability.

C9-grafted particles (Figure 8c1,d1) exhibit a markedly stronger toluene adsorption and reduce water penetration, consistent with enhanced hydrophobicity and greater experimental emulsion stability. The longer alkyl chains also display distinct tilting toward the oil phase, further facilitating particle anchoring at the interface.

Local concentration profiles (Figure 8a2–d2) were used to quantify the extent of interfacial nanomixing (gray zones). For C3-Janus particles, the local toluene and water concentrations (0.48 and 38 mol L^–1^ at 26 Å) yield a W/T molar ratio of 79:1—significantly higher miscibility than in the bulk (623:1), with toluene and water concentrations of 0.098 and 60.96 mol/L (at 36 Å), respectively. Homogeneous C3 particles exhibit weaker nanomixing (104:1), corresponding to local water and toluene densities of 0.38 and 40 mol/L (at 26 Å), respectively. The bulk concentrations of toluene and water are 0.146 and 59.012 mol/L (at 33 Å), respectively, with a W/T molar ratio of 405:1. Comparison of these ratios confirms higher water–toluene miscibility at the particle surface, though to a lesser extent than that on C3-grafted Janus particles. C9-Janus particles achieve the strongest nanomixing effects (13:1) for local toluene and water densities of 2.1 and 27 mol/L (at 26 Å), respectively, far exceeding their homogeneous analogues (18:1) with 1.7 and 30 mol/L densities (at 33 Å), respectively.

Collectively, these results reveal that Janus particles, particularly those grafted with C9 chains, not only stabilize emulsions more effectively but also promote local toluene–water nanomixing at the particle interface. This nanoscale miscibility underpins their superior interfacial stabilization capacity, providing a mechanistic bridge between molecular-scale interactions and macroscopic emulsion behavior.

## Conclusions

By integrating experimental observations with multiscale simulations (classical MD and DPD), we elucidated how the density, length, and spatial distribution of surface-grafted silanes govern the formation and stability of toluene/water Pickering emulsions. The combined results reveal clear structure–property relationships at the nanoscale: increasing the alkyl chain length enhances emulsion stability, as longer hydrophobic ligands achieve strong interfacial anchoring at lower surface densities. Beyond a critical chain length (≈C9), further elongation yields no additional stabilization, indicating a saturation of the interfacial adsorption efficiency.

Consistent with these findings, molecular dynamics simulations show that shorter chains (C3) interact predominantly with water, whereas longer chains (C9) enable partial penetration of toluene molecules, leading to enhanced local nanomixing and stronger oil–water interfacial cohesion that correlates with enhanced emulsification. Moreover, the particle architecture plays a decisive role: Janus particles consistently produce more stable emulsions than their homogeneously functionalized counterparts owing to a broader stability window and more favorable interfacial energetics. These computational trends align closely with the experimental emulsification data.

Overall, this study demonstrates how the synergy between experiments and multiscale simulations can accelerate the rational design of surface-active particles for interfacial applications. The insights gained here provide guiding principles for tuning the wettability, interfacial adsorption, and nanoscale mixing in particle-stabilized emulsions. We anticipate that this combined approach will be equally valuable for understanding and engineering more complex systems, such as particle-stabilized foams and multiphase catalytic interfaces, where nanoscale organization dictates macroscopic behavior.

## Supplementary Material



## Data Availability

All data is provided in full in the results section of the manuscript and Supporting Information.

## Notes

Simulation details on DPD and MD methods, including methods for computing interfacial tension between water and toluene (DPD) and local concentration profiles (MD); conservative interaction parameters (*a_ii_* and *a_ij_*) for DPD simulations; representation of the water–toluene system used to parametrize the DPD force field; snapshots of simulations used to obtain the *a_ij_* parameters for silica, OH, and propane (red) beads; representation of the water–toluene system used to parametrize the DPD force field; definition of the 3-phase contact angle, θ_12_, including the contribution of the line tension; snapshots of DPD simulations after 50 ns; list of experimental methods for particle synthesis, characterization, and emulsification protocols; TGA profiles of pristine silica particles, propyltriethoxysilane-modified silica particles, and octyltriethoxysilane-modified silica particles; HR-TEM micrographs of pristine and modified silica particles; size distribution of emulsion droplets stabilized by different concentrations of C3-JP-10 particles; weight loss of pristine silica particles, propyltriethoxysilane, and octyltriethoxysilane-grafted Janus and homogeneous particles at different temperature ranges; list of main properties of particles prepared in this study; particle adsorption energies and free energies of droplet formation (PDF)
