# Supplementary material for: Multiscale Insights into the Genesis of Pickering Emulsions: Nanomixing and Interfacial Design of Surface-Active Silica Particles
Source: Langmuir. 2026 Mar 4;42(10):7136–44. doi: 10.1021/acs.langmuir.5c05703 (PMC13001096; doi:10.1021/acs.langmuir.5c05703)
Supplement: Supplementary file 1 [file la5c05703_si_001.pdf]

Supporting Information

Multiscale Insights into the Genesis of Pickering Emulsions: Nanomixing and Interfacial Design of Surface-Active Silica Particles

Kang Wang, Antoni Salom-Català, Alberto Roldan*, Marc Pera-Titus*

Cardiff Catalysis Institute, Translational Research Hub, Cardiff University, Maindy Road, Cardiff, CF24 4HQ

* *Corresponding author:* roldanmartineza@cardiff.ac.uk *(ARM),* peratitusm@cardiff.ac.uk *(MPT)*

Number of pages: 21

Number of figures: 7

Number of tables: 3

TABLE OF CONTENTS:

**S1. SIMULATION DETAILS**	**S4**
S1.1. Dissipative particle dynamics (DPD)	S4
S1.2. Parametrization	S5
S1.3. Computational details	S7
S1.4. Computation of interfacial tension between water and toluene in DPD simulations	S7
S1.5. Calculation of the local concentration profiles	S8
**S2. EXPERIMENTAL SECTION**	**S8**
S2.1 Reagents and materials	S8
S2.2. Preparation of pristine silica particles	S8
S2.3. Preparation and characterization of alkyl-grafted silicas	S8
S2.3.1. Janus silica particles	S8
S2.3.2. Preparation of homogeneous silica particles	S9
S2.4. Characterization techniques	S9
S2.5. Emulsification studies	S10
S2.6. Effective surface area of particles	S10

GLOSSARY **S18**

REFERENCES **S20**

FIGURES

**Figure S1.** Representation of the water-toluene system used to parameterize the DPD force field. Calculated and experimental values (in brackets) for the density (ρ) and interfacial tension (γ) calculated as shown in section S1.3 are also shown.	S6
**Figure S2.** Snapshots of simulations used to obtain the a_{ij} parameters for silica (yellow), OH (green) and propane (red) beads. Blue and grey beads are water and toluene beads respectively. The fluids have changed positions to keep the nano-particle at the center of the box for the sake of clarity.	S6
**Figure S3.** Definition of the 3-phase contact angle, θ_{12}, including the contribution of the line tension (τ). Phase 2 (most polar phase) is considered as reference. The contact line is labeled in red. Unlike surfactants, particles do not affect the emulsion stability by significantly reducing $\gamma_{1/2}$. Besides, particle adsorption encompasses a concomitant swelling of droplets/bubbles by an amount dependent on the particle radius, *r*, and the contact angle, θ_{12}. A similar analysis can be carried out for Janus particles with an asymmetric distribution of hydrophilic / hydrophobic groups, but defining different surface tensions for the polar (P) and apolar (A) regions of the particles and phases, i.e. $\gamma_{P/1}$, $\gamma_{P/2}$, $\gamma_{A/1}$ and $\gamma_{A/2}$.	S11
**Figure S4.** Snapshots DPD of simulations after 50 ns with an initial point corresponding to a completely separated solvent phases.	S12
**Figure S5.** TGA profiles of (a) pristine silica particles. (b) propyltriethoxysilane-modified silica particles. (c) octyltriethoxysilane-modified silica particles.	S13
**Figure S6.** HR-TEM micrographs of pristine and modified silica nanoparticles. a) Pristine silica, b) C3-JP-10, c) C3-HP-10.	S14
**Figure S7.** Size distribution of emulsion droplets stabilized by different concentrations of C3-JP-10 particles: (a) 0.5 wt%; (b) 0.75 wt%; (c) 1.0 wt%; and (d) 1.5 wt%. Emulsification conditions: 25 ºC, 1:1 toluene/water volume ratio, 0.5 wt%, 0.75 wt%, 1.0 wt%, and 1.5 wt% C3-JP-10 particle loading, homogenization at 30,000 rpm for 15 s.	S15

TABLES

**Table S1**. Conservative interaction parameters (a_{ii} and a_{ij}) used in this work for the DPD simulations in units k_BT/r_c.	S6
**Table S2.** Weight loss of pristine silica particles, propyltriethoxysilane and octyltriethoxysilane grafted Janus (JP) and homogeneous (HP) particles at different temperature ranges.	S16

**Table S3.** List of main properties of particles prepared in this study, particle adsorption energies and free energies of droplet formation ($\Delta G_{droplet}$)	S17

S1. SIMULATION DETAILS

S1.1. Dissipative particle dynamics (DPD)

In DPD simulations, the bead motion is described by the Newton's equation of motion. The external force ($\mathbf{F_i}$) of each bead is composed of three components: conservative force ($\mathbf{F_{ij}}^{\mathbf{C}}$), dissipative force ($\mathbf{F_{ij}}^{\mathbf{D}}$), and random force ($\mathbf{F_{ij}}^{\mathbf{R}}$).[1,2,3]

$$\mathbf{F}_{\mathrm{i}} = \sum_{i\neq j}\left(\mathbf{F}_{\mathrm{ij}}^{\mathrm{C}} + \mathbf{F}_{\mathrm{ij}}^{\mathrm{D}} + \mathbf{F}_{\mathrm{ij}}^{\mathrm{R}}\right), \qquad \text{(S1)}$$

The conservative force ($\mathbf{F_{ij}}^{\mathbf{C}}$) is expressed by the contribution of the soft interaction force, and is also the primary force over the bead. This force can be accounted by the expression

$$\mathbf{F}_{\mathrm{ij}}^{\mathrm{C}} = \begin{cases} a_{\mathrm{ij}}\left(1 - r_{\mathrm{ij}}/r_{\mathrm{c}}\right)\hat{\mathbf{r}}_{\mathbf{ij}} & r_{\mathrm{ij}} < r_{\mathrm{c}} \\ 0 & r_{\mathrm{ij}} \geq r_{\mathrm{c}}, \end{cases} \qquad \text{(S2)}$$

where $r_{ij} = r_i - r_j$ is the centroid distance between the ith and j^{th} beads, $\hat{\mathbf{r}}_{\mathbf{ij}} = r_{ij}/|r_{ij}|$ is the unit vector of the direction from the ith to the jth bead, r_C is the cut-off radius of the pairwise bead interaction, setting the basic length-scale in DPD simulations, and the coefficient a_{ij} represent the maximum repulsion between two interacting beads. When bead types are the same ($i = j$), the interaction parameter between the same bead type (a_{ii}) can be expressed as follows[4]

$$a_{\mathrm{ii}} = \frac{75\, k_B T}{\rho}, \qquad \text{(S3)}$$

where ρ is the bead number density, k_B is the Boltzmann constant, T is the absolute temperature and 75 is a constant value determined by Groot and Warren[1] when they formulated the DPD method to match the compressibility of water. k_BT represents the reduced energy in DPD simulations and was set to 1.

Parameters a_{ii} must be calculated to calculate parameters a_{ij}. As proposed by Groot and Warren, a_{ii} is the same for all the intraspecies interactions involved in the system. When the bead number density of the system is 3, the intra-species interactions (a_{ii}) is 25.[4,5] Moreover, if the isothermal compressibility for water is used to calculate a_{ii} as proposed by Groot and Warren, the value obtained is, in fact, 25. However, some other authors reported that this value should be related with the coarse-graining degree of the water molecule. For instance, Groot and Rabone reported that, since in their work one water bead represents 5 water molecules, a_{ii} should be 131.5.[6] In our case, after preliminary simulations to validate the model, we found that the value of a_{ii} = 85 worked for our system to match the results of the calculations with the experimental values of density and interfacial tension (**Figure S1**).

The inter-species interaction parameters (a_{ij}) can be calculated from the Flory-Huggins binary interaction parameters (χ_{ij}) as a function of ρ[4]

$$\Delta a_{\mathrm{ij}} = a_{\mathrm{ij}} - a_{\mathrm{ii}} = \chi_{\mathrm{ij}}/0.286 \quad (\rho\, r_{\mathrm{c}}^3 = 3); \qquad \text{(S4)}$$

$$\Delta a_{\mathrm{ij}} = a_{\mathrm{ij}} - a_{\mathrm{ii}} = \chi_{\mathrm{ij}}/0.689 \quad (\rho\, r_{\mathrm{c}}^3 = 5). \qquad \text{(S5)}$$

The Flory-Huggins parameters can be computed from the Hansen solubility parameters[7] using the expressions

$$\chi_{ij}(T) = \frac{V_{ij}}{RT}\left[\delta_i(T) - \delta_j(T)\right]^2, \tag{S6}$$

$$\delta_i^2 = \delta_{i,d}^2 + \delta_{i,p}^2 + \delta_{i,hb}^2, \tag{S7}$$

where δ_i is the Hansen solubility parameter, and $\delta_{i,d}$, $\delta_{i,p}$, and $\delta_{i,db}$ represent the dispersion, polar, and hydrogen-bonding components,[8] V_{ij} is the partial molar volume of the DPD bead, and R is the constant of perfect gases.

In addition to the conservative force, the dissipative force ($\mathbf{F_{ij}^D}$) and the random force ($\mathbf{F_{ii}^R}$) represent the viscosity contribution and the thermal fluctuation contribution of system, respectively. These forces can be expressed as follows

$$\mathbf{F}_{ij}^{D} = -\eta\omega^{D}(r_{ij})(\mathbf{\hat{r}_{ij}} \cdot \mathbf{v_{ij}})\,\mathbf{\hat{r}_{ij}}; \tag{S8}$$

$$\mathbf{F}_{ij}^{R} = \sigma\omega^{R}(r_{ij})\zeta_{ij}\,\Delta t^{-1/2}\,\hat{r}_{ij}. \tag{S9}$$

where η is the friction coefficient, $\boldsymbol{v_{ij}} = \boldsymbol{v_i} - \boldsymbol{v_j}$ is the relative velocity between the ith and j^{th} beads, $\boldsymbol{\sigma}$ is the amplitude of noise, ξ_{ij} is a random number with a zero mean and unit variance with a the Gaussian distribution, Δt is the time step, and $w^D(r_{ij})$ and $w^R(r_{ij})$ are the weight functions for dissipative and random forces, which are related by the expression

$$\omega^{D}(r_{ij}) = \left[\omega^{R}(r_{ij})\right]^2 = \begin{cases} a_{ij}\left(1 - r_{ij}/r_c\right)^2 & r_{ij} < r_c \\ 0 & r_{ij} \geq r_c \end{cases} \tag{S10}$$

$$\sigma = \sqrt{2\eta k_B T} \tag{S11}$$

Along this work, we used reduced DPD units to boost the calculations. We used r_C and k_BT as length and energy units, respectively. Besides, the mass of the beads, m, was set equal to 1. In this study, r_c was set to 8.14 Å.

S1.2. Parametrization

We started the parameterization for a pure water-toluene biphasic system without particles. We used a box of 15 x 15 x 30 Å^3 filled with 20250 beads in total 10125 beads for each, water and toluene to have a 1:1 w/t ratio in beads. This means a ratio of 1:6 w/t in molecules. Due to the lack of consensus in the literature, we used different starting values for a_{ii}, and using Eq 2 and 3, we calculated the water-toluene interaction parameter. The value which gave a good behaviour, that is the calculated densities and interfacial tension matched with the experimental values, of the system is shown in **Table 1** using reported The Hansen solubility parameters.[9] A representation of the system using the calculated value, with some critical parameters such as the interfacial tension and the density is shown in **Figure S1**.

With this value, we calculated the a_{ij} for the rest of the beads involved in the simulations (silica, OH and propane). To validate those values, we performed two different simulations. The first one with a nanoparticle formed of a silica core and completely covered by hydrophilic groups. In the second simulation, the nanoparticle was covered only by hydrophobic groups. Two snapshots of these simulations are shown in **Figure S2**. In both simulations, the behavior is what one should expect. In the case of the hydrophilic nanoparticle, it remains in the water phase, while in the case of the hydrophobic particle, it remains in the oil phase. These results reinforced the

choice of values in the parameterization (**Table 1**), and these were further implemented to run the simulations in our study.

Table S1. Conservative interaction parameters (a_{ii} and a_{ij}) used in this work for the DPD simulations in units k_BT/r_c.

	Water	Toluene	Silica	OH	Propane
**Water**	85.0				
**Toluene**	151.7	85.0			
**Silica**	114.1	95.8	85.0		
**OH**	70.0	166.7	114.1	85.0	
**Propane**	177.0	70.7	100.6	159.0	85.0

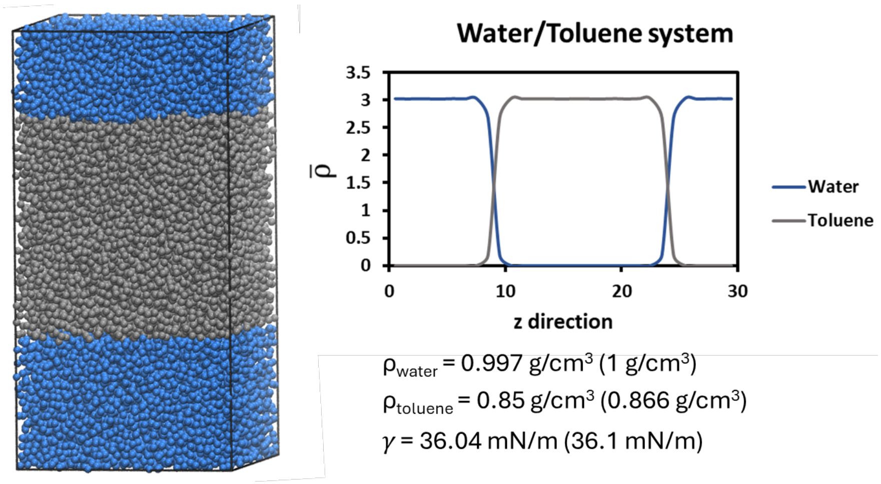

Figure S1. Representation of the water(blue)-toluene(grey) system used to parametrize the DPD force field. Calculated and experimental values (in brackets) for the density (ρ) and interfacial tension (γ) calculated as shown in section S1.3 are also shown.

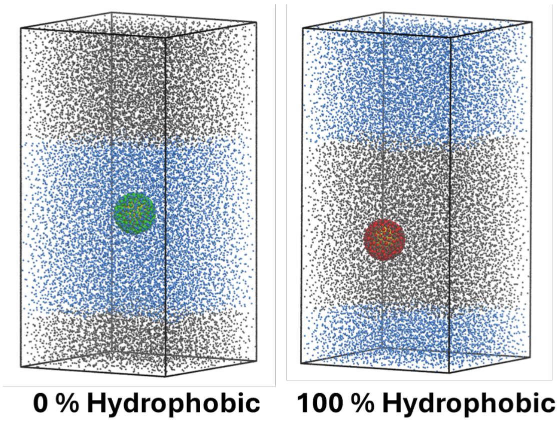

Figure S2. Snapshots of simulations used to obtain the a_{ij} parameters for silica (yellow), OH (green) and propane (red) beads. Blue and grey beads are water and toluene beads respectively. The fluids have changed positions to keep the nanoparticle at the center of the box for the sake of clarity.

S1.3. Computational details

DPD. For the DPD simulations we used the reduced units to make the resolution of the equations easier for such a big system. That means that the distance, energy and mass were set at 1 r_c, 1 k_BT and 1 m, respectively. The timestep was set at 0.01τ. The translation to real units for all these magnitudes are r_c = 8.14 Å, T = 298 K, and timestep = 3.27 ps. The simulation box size was 15x15x30 r_c^3. The total number of steps was set to 2000000. The solvent mixture was formed by a 1:1 ratio with a total number of solvent beads of 20250. Note that the total number of beads are the sum of solvent beads plus the nanoparticle beads commented above. All simulations were carried out within an NVE ensemble. Lammps software (version 5 June 2019) was used to perform the calculations.[10] The constants for the dissipative and random forces were set at η = 4.5 and σ = 3 to keep the temperature constant at k_BT = 1.

Classical MD. The version 5 June 2019 of Lammps software was used to carry out all classical MD simulations. The box size was 66.734 x 34.842 x 100 Å^3. The number of toluene and water molecules were set at 482 and 2849 for a 1:1 v/v mixture matching the experimental densities, i.e. 0.865 and 1 g/cm^3, respectively, considering the space not occupied by the slab in the simulation box. The COMPASS force field developed by H. Sun[11] was used to define the bonding and non-bonding interactions. The distance cutoff for the Lennard-Jones and coulomb interactions was set to 12 Å. All parameters describing the slab were taken from the work of Emami and co-workers.[42] Regarding the silane groups, all parameters were taken from ref. [12.] The parameters for water were those reported by Pathirannahalage et al.[13] For toluene, the bonding and Lennard-Jones parameters were taken from the original work of COMPASS force field, while the charges were calculated in-house using Gaussian software.[14] We used Mulliken charges calculated within the Density Functional Theory (DFT) framework[15] using the B3LYP functional.[16,17,18] For all atoms we used a standard 6-31G(d,p) basis set.[19,20,21] A GD3 dispersion correction was also applied.[22] The timestep was 1.0 fs. The equilibration protocol was the following: 1) energy minimization with an energy and a force threshold of 10^{-4} kcal/mol and 10^{-6} kcal/mol A^{-1}, respectively; 2) 500 ps of NVT ensemble; 3) 500 ps of NPT simulation; 4) 500 ps of NVT; and finally 5) 50 ns of NVT considered the production runs, where the last 25 ns of simulation were taken for analysis. The temperature and pressure were set at 298 K and 1 atm, respectively. The Nose-Hoover thermo- and barostat were used to perform the time integration of the equations of motion.

S1.4. Computation of the interfacial tension between water and toluene in DPD simulations

The interfacial tension (γ) of the water-toluene interface for DPD parameterization and validation was calculated using the following expression:[23,24]

$$\gamma = \frac{L_z}{2}\left(P_{zz} - \frac{P_{xx} + P_{yy}}{2}\right); \quad \text{(S12)}$$

where L_z is the box length in the z direction and P_{xx}, P_{yy} and P_{zz} are the pressures in the x, y and z directions, respectively. L_z is divided by 2 because there are two water-toluene interfaces presents in the system (**Figure S1**)

S1.5. Calculation of the local concentration profiles

First, the simulation box was divided into 100 slices in the XY-plane direction along the Z direction and the concentrations of water and toluene were averaged. Secondly, we plotted the averaged concentrations of each slice along the Z direction of the box. The water and toluene concentrations were calculated separately, and this procedure was repeated for all four systems studied. The interface was set to be the length of the ligands grafted on the surface.

The W/T ratios were calculated dividing the local concentrations of water and toluene at 26 Å in the Z direction, that corresponds to the middle of the interface in the C_3 systems where the concentration of toluene is maximum. For the C_9 systems we selected the same point of analysis for the sake of comparison.

S2. EXPERIMENTAL SECTION

S2.1 Reagents and materials

Tetraethyl orthosilicate (TEOS, 98%), Aqueous ammonia solution (28-30%), anhydrous ethanol (99.9%), all purchased from Sigma-Aldrich, were used for the synthesis of silica particles. Paraffin wax (melting point: 65 °C), n-cetytrimethylammonium bromide (CTAB, 98%), n-propyltriethoxysilane (97%), n-octyltriethoxy silane (97%), and toluene (99.5%) also supplied by Sigma-Aldrich, were used for preparation of Janus silica particles and emulsification test. Deionized water was produced by the Elga PURELAB ultrapure water machines.

S2.2. Preparation of pristine silica particles

The synthesis of silica particles was carried out by the Stöber method, whereby a mixture of 15 mL of water, 50 mL of ethanol and 3 mL of 35% of an aqueous ammonia solution was heated to 25 °C. Subsequently, 6 mL of 99% TEOS dissolved in 50 mL ethanol was added to this solution and rapidly stirred at a rate of 1500 rpm for 1 min to form the silica seeds. The reaction was then allowed to proceed for 60 min at 500 rpm. The resulting silica spherical particles were separated by centrifugation and washed three times with ethanol and water before drying at 110 °C in air overnight. The particles are denoted as pristine silica.

S2.3. Preparation and characterization of alkyl-grafted silicas

S2.3.1. Janus silica particles

Initially, 400 mg of the pristine silica particles were dispersed in 30 mL of an aqueous CTAB solution (0.45 mM), followed by the addition of 4.5 g of paraffin wax at 75 °C at constant stirring (300 rpm) until all the wax was completely melted. The resulting emulsion was formed using an ultra-turrax at 30,000 rpm for 1 min, followed by cooling to room temperature, causing the paraffin wax to solidify. Subsequently, the wax-in-water emulsions were washed with deionized water with 1 L deionized water to eliminate all the particles in the aqueous phase, weakly attached particles on the wax droplets, and CTAB molecules. The filter cake was termed SiO_2@Wax.

C3-Janus particles: SiO_2@Wax was redispersed into 0.3 mL of an aqueous ammonia solution (28-30 wt%) in 15 mL of deionized water and was reacted with 10 or 20 µL of n-propyltriethoxysilane for 1.5 h at room temperature to modify the exposed surfaces. The resulting wax droplets

were filtered, washed with water on the filter, and dried overnight at 25 °C. Subsequently, the wax was dissolved in cyclohexane, and the particles were washed 3 times using cyclohexane followed by centrifugation, and dried overnight at 110 °C. The particles reacted with 10 and 20 μL of n-propyltriethoxysilane were termed as C3-JP-10 and C3-JP-20, respectively.

C8-Janus particles: SiO_2@Wax was redispersed into 0.3 mL of an aqueous ammonia solution (28-30 wt%) in 15 mL of deionized water and was reacted with 10 or 20 μL of n-octyltriethoxysilane for 50 min at room temperature to modify the exposed surfaces. The resulting wax droplets were filtered, washed with water on the filter, and dried overnight at 25 °C. Subsequently, the wax was dissolved in cyclohexane, and the particles were washed 3 times using cyclohexane followed by centrifugation and dried overnight at 110 °C. The particles that reacted with 10 and 20 μL of n-octyltriethoxysilane were termed as C8-JP-10 and C8-JP-20, respectively.

S2.3.2. Preparation of homogeneous silica particles

C3-grafted homogeneous particles: 400 mg of pristine silica particles were dispersed in 40 mL of deionized water in a 100 mL round bottom flask, and 0.3 mL of an aqueous ammonia solution (28-30 wt%) were added. Then, 10 or 20 μL of n-propyltriethoxysilane were added to the suspension at room temperature and stirred for 1.5 h. The suspension was next filtered with deionized water and the filter cake was dried overnight at 110 °C. The particles reacted with 10 and 20 μL of n-propyltriethoxysilane were termed as C3-HP-10 and C3-HP-20, respectively.

C8-grafted homogeneous particles: 400 mg of pristine silica particles were dispersed in 15 mL of deionized water in a 100 mL round bottom flask, and 0.3 mL of an aqueous ammonia solution (28-30 wt%) were added. Then, 10 or 20 μL of n-octyltriethoxysilane were added to the suspension at room temperature and stirred for 50 min. The suspension was next filtered with deionized water and the filter cake was dried overnight at 110 °C. The particles that reacted with 10 or 20 μL of n-octyltriethoxysilane were termed as C8-HP-10 and C8-HP-20, respectively.

S2.4. Characterization techniques

Thermogravimetric analysis (TGA) was carried out on a PerkinElmer TGA 4000 Instrument. The particles (~10 mg in 100-μL alumina crucible) were treated from 30 to 900 °C with a heating rate of 10 °C/min under airflow of 30 mL(STP)/min.

Brunauer-Emmett-Teller (BET) surface area measurements of silica particles were performed using a Quadrasorb surface area analyzer. The specific surface area of a given particle sample was measured through a five-point nitrogen (N_2) adsorption isotherm. Prior to analysis, the samples were degassed at 130 °C for 3 h to remove any adsorbed impurities. Surface area calculations were conducted at -196 °C based on the five-point N_2 adsorption data, employing the BET method for analysis.

The particle size distributions of silica particles were measured by TEM on a JEOL JEM-2100 microscope operating at 200 kV. Samples were prepared by dry deposition onto 300 mesh copper grids coated with holey carbon film. The images were analyzed by ImageJ software. At least 100 particles were counted to measure particle size distributions.

The hydrophilic-lipophilic balance (HLB) of the particles was measured as the molar ratio between the molar number (n) of hydrophilic groups (SiOH) and molar number of lipophilic groups (alkyl chains) as follows

$$\mathrm{HLB} = \frac{n_{Si-OH}}{n_{alkyl\ chains}} \tag{S13}$$

S2.5. Emulsification studies

Emulsions for the water-toluene system were prepared in a 7-mL sealable vial at room temperature under vigorous homogenization (30,000 rpm, ultra-turrax IKA T-10) for 15 s with a 1:1 water/toluene volume ratio at different particle concentrations. The emulsions were stored at room temperature to check their stability over time. The emulsion volume was determined by direct inspection of the toluene/water emulsion using a Nikon D300s camera equipped with a macro lens. The emulsion height was measured by direct inspection of the emulsion using a ruler. The morphology of emulsion droplets stabilized by modified silica particles were inspected by Leica DM750 optical microscope with GXCAM software, 10× ocular, 4×, 10×, 40×, and 100× objectives were used to measure the bubble size. ImageJ software was used to quantify average droplet sizes.

S2.6. Effective surface area of particles

The representation of the inverse of the mean droplet size against the weight fraction of particles in a Pickering emulsion shows a linear trend for particle loadings lower than the critical mass fraction (CMF). We can rationalize and extract relevant information from this linear trend using a simple model based on a reduced number of assumptions.

Let us consider a droplet stabilized by an ideal film of nonporous particles at the toluene/water interface. Assuming that the adsorbed particles arrange in a configuration minimizing the interfacial energy in a similar manner as proposed by Kraft *et al*,[25] the mean droplet size for an emulsion based on the toluene-water system can be estimated using the general expression:

$$\Phi\, X_D\, \bar{S}_D = X_p\, \bar{S}_{p,eff} \text{ for } X_p < \mathrm{CMF} \tag{S14}$$

where X_D and X_p are the weight fraction of the disperse phase and particles, respectively, $\bar{S}_D$ and $\bar{S}_{p,eff}$ are the specific and effective surface areas, and Φ is the particle coverage at the water-toluene interface.

The average droplet diameter, $\bar{D}$, can be written as a function of the effective surface area of the particles as follows[26,27,28]

$$\frac{1}{\bar{D}} = \frac{\bar{S}_{p,eff}}{\Phi}\frac{\rho}{6}\frac{X_p}{X_D} \text{ for } X_p < \mathrm{CMF} \tag{S15}$$

where ρ is the density of the disperse phase.

The surface density of adsorbed particles, Γ_p, was calculated by dividing the total number of particles, N_p, by the interfacial surface area. The following expression was used

$$\Gamma_p = \frac{N_p}{S_{int}} = \frac{3}{4}\frac{(V_D\,\rho_D + V_C\,\rho_C)}{\rho_{SiO_2}\,\pi r^3}\frac{}{S_{int}} \text{ for } X_p < \mathrm{CMF} \tag{S16}$$

where V_D and V_C are the volumes of the disperse and continuous phase, respectively (1 mL in each case), ρ_D and ρ_C are corresponding densities (0.867 g.cm^{-3} and 1.0 g.cm^{-3}, respectively), and ρ_{SiO2} and r are the density (2.2 g.cm^{-3}) and average radius of particles.

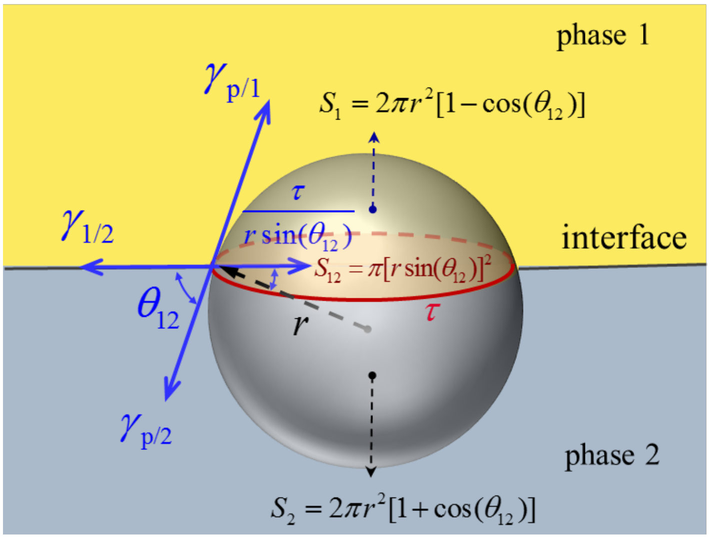

Figure S3. Definition of the 3-phase contact angle, θ_{12}, including the contribution of the line tension (τ). Phase 2 (most polar phase) is considered as reference. The contact line is labeled in red. Unlike surfactants, particles do not affect the emulsion stability by significantly reducing $\gamma_{1/2}$. Besides, particle adsorption encompasses a concomitant swelling of droplets/bubbles by an amount dependent on the particle radius, r, and the contact angle, θ_{12}. A similar analysis can be carried out for Janus particles with an asymmetric distribution of hydrophilic / hydrophobic groups, but defining different surface tensions for the polar (P) and apolar (A) regions of the particles and phases, i.e. $\gamma_{P/1}$, $\gamma_{P/2}$, $\gamma_{A/1}$ and $\gamma_{A/2}$. Image adapted from ref.[29]

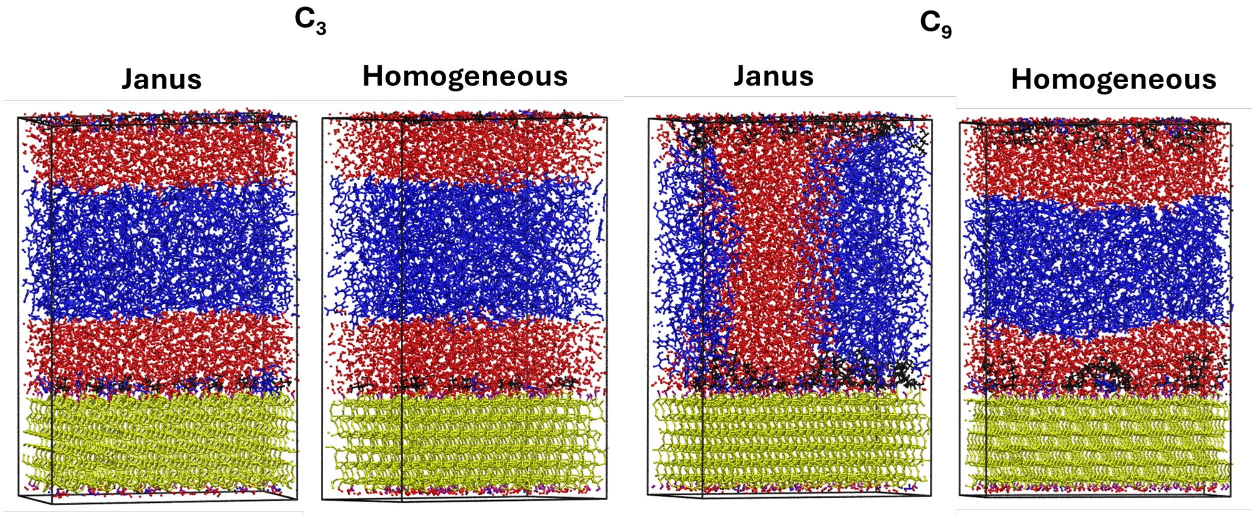

Figure S4. Snapshots of DPD simulations after 50 ns with an initial point corresponding to a completely separated solvent phases.

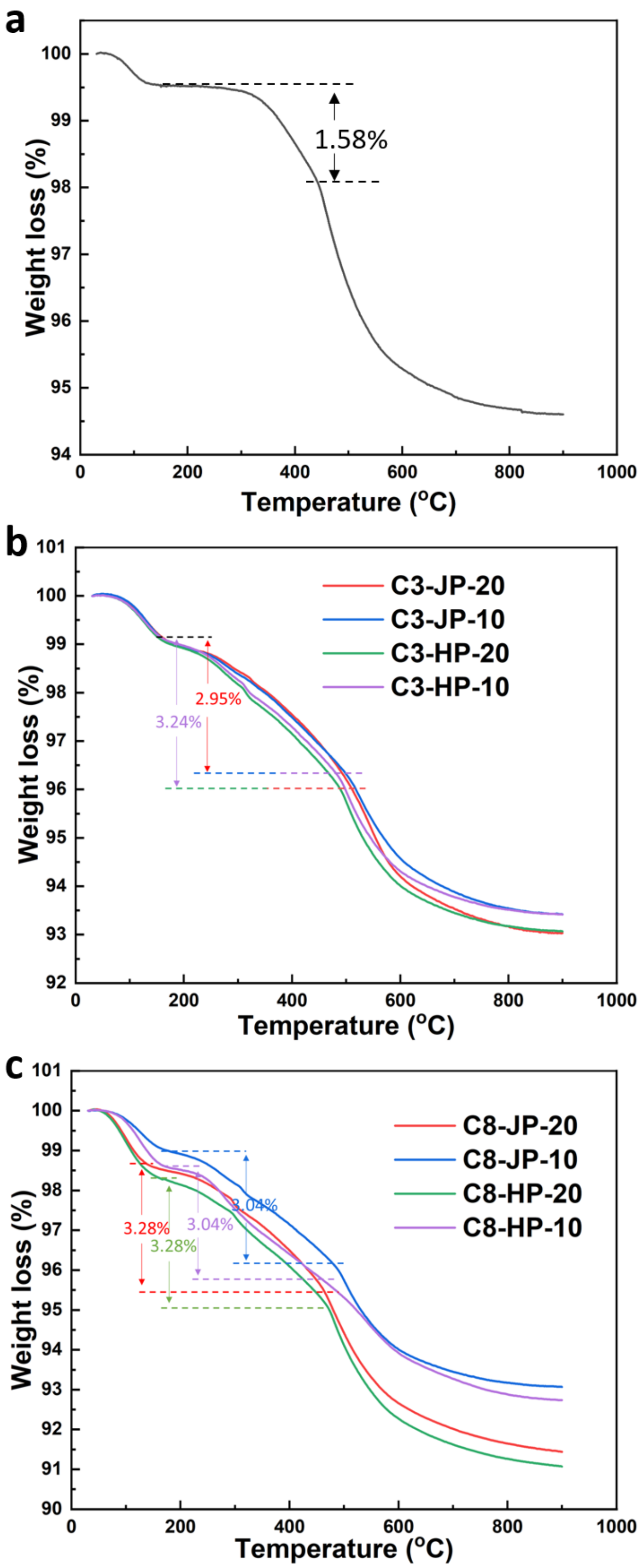

Figure S5. TGA profiles of (a) pristine silica particles. (b) propyltriethoxysilane-modified silica particles. (c) octyltriethoxysilane-modified silica particles.

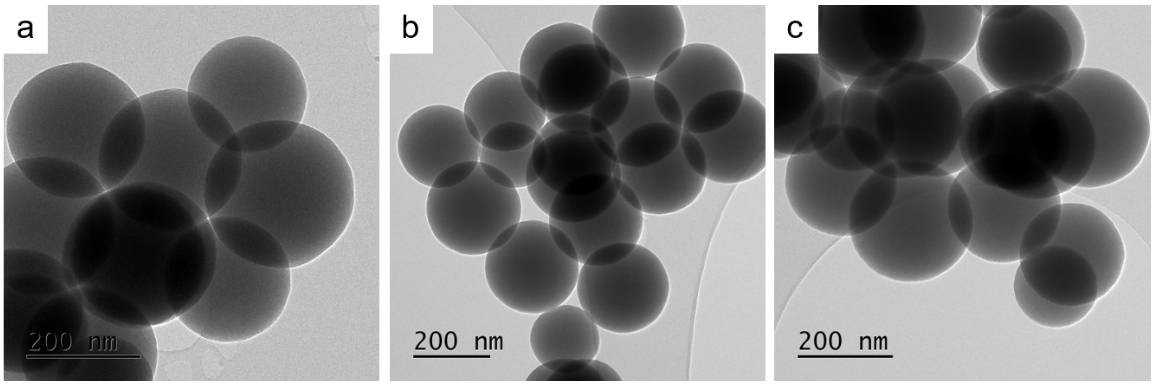

Figure S6. HR-TEM micrographs of pristine and modified silica nanoparticles. a) Pristine silica, b) C3-JP-10, c) C3-HP-10.

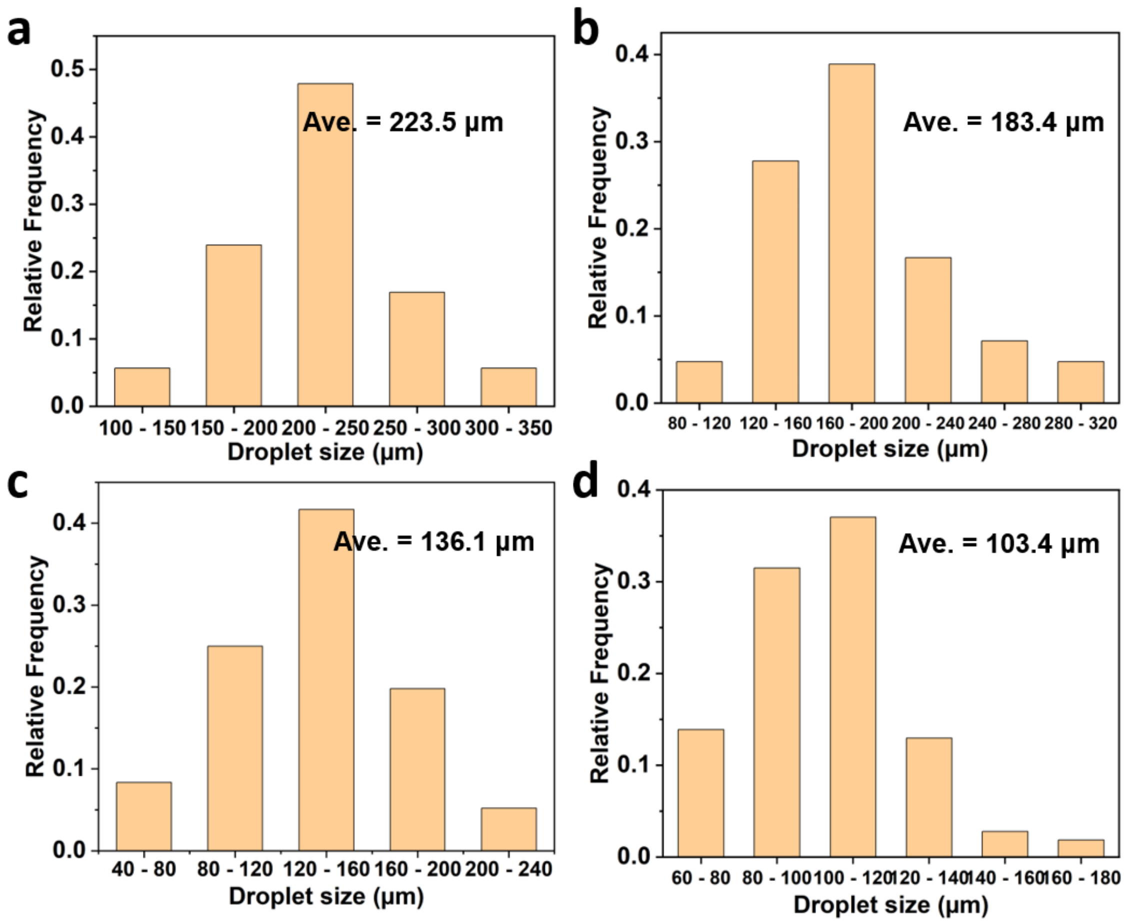

Figure S7. Size distribution of emulsion droplets stabilized by different concentrations of C3-JP-10 particles: (a) 0.5 wt%; (b) 0.75 wt%; (c) 1.0 wt%; and (d) 1.5 wt%. Emulsification conditions: 25 ºC, 1:1 toluene/water volume ratio, 0.5 wt%, 0.75 wt%, 1.0 wt%, and 1.5 wt% C3-JP-10 particle loading, homogenization at 30,000 rpm for 15 s.

Table S2. Weight loss of pristine silica particles, propyltriethoxysilane and octyltriethoxysilane grafted Janus (JP) and homogeneous (HP) particles at different temperature ranges.

Entry	Particle	Weight loss (30-150 °C)	Weight loss (150-450 °C)	Weight loss (450-900 °C)	Density (chains/nm^2)	Density (SiOH/nm^2)
1	Pristine	0.46%	1.58	3.46	-	70.0
2	C3-JP-20	0.86%	3.24	2.87	27.0	43.0
3	C3-JP-10	0.86%	2.95	2.77	24.6	45.4
4	C3-HP-20	0.86%	3.24	2.82	27.0	43.0
5	C3-HP-10	0.86%	2.95	2.77	24.6	45.4
6	C8-JP-20	0.32%	3.28	3.96	11.1	58.9
7	C8-JP-10	0.91%	3.04	2.98	10.3	59.7
8	C8-HP-20	0.69%	3.28	3.96	11.1	58.9
9	C8-HP-10	0.38%	3.04	2.84	10.3	59.7

Table S3. List of main properties of particles prepared in this study, particle adsorption energies and free energies of droplet formation ($\Delta G_{droplet}$)

Particle	C3-JP-10	C3-HP-10	C3-JP-20	C3-HP-20	C8-JP-10	C8-HP-10	C8-JP-20	C8-HP-20
Volume dispersed phase (mL)	1.0	1.0	1.0	1.0	1.0	1.0	1.0	1.0
Mass of particles (mg)	9.25	9.25	9.25	9.25	9.25	9.25	9.25	9.25
Droplet diameter (μm)	224 ± 28	302 ± 30	251 ± 25	331 ± 27	231 ± 25	289 ± 27	242 ± 20	322 ± 22
S_{int} (cm^2)	268	199	239	181	260	208	248	186
Particle diameter (nm)	253	253	253	253	253	253	253	253
Number of particles ($x10^{11}$)	4.96	4.96	4.96	4.96	4.96	4.96	4.96	4.96
Particle density (NP/μm^2)	18.51	24.96	20.74	27.35	19.09	23.88	20.00	26.61
Ψ (-)	0.93	1.25	1.04	1.38	0.96	1.20	1.01	1.34
Φ (-)	0.92	1.17	1.02	1.29	0.64	0.43	0.54	0.48
Angle (°)	85.0	105.0	81.0	104.0	125.0	143.0	133.0	143.0
**Ed (J)**	**1.51 $x10^{-15}$**	**9.94 $x10^{-16}$**	**1.29 $x10^{-15}$**	**1.04 $x10^{-15}$**	**3.29 $x10^{-16}$**	**7.34 $x10^{-17}$**	**1.83 $x10^{-16}$**	**7.34 $x10^{-17}$**
**$\Delta G_{droplet}$ (nJ)**	**1.2 ± 0.2**	**3.1 ± 0.08**	**1.8 ± 0.2**	**2.5 ± 0.4**	**5 ± 1**	**9 ± 1**	**6.0 ± 0.9**	**11 ± 1**

GLOSSARY

a_{ij}	Maximum repulsion between the ith and j^{th} beads [$J.m^{-1}$]
d	Distance between the SiNP center and the W/O interface [m]
$\bar{D}$	Average diameter [m]
E	Interfacial adsorption energy of SiNPs [$J.mol^{-1}$]
$\mathbf{F_i}$	Total force exerted on the ith bead [N]
$\mathbf{F_{ij}}$	Force between the ith and j^{th} beads [N]
k_B	Boltzmann constant ($1.38x10^{-23}$ $J.K^{-1}$)
k_S	Spring constant [$N.m^{-1}$]
L_i	Length of the simulation box along the ith direction [m]
m_i	Mass of the ith bead [kg]
n	Number of beads in the hydrophobic polymers [-] or number of moles [-]
N_p	Number of SiNPs [-]
P	Pressure tensor [Pa]
r_c	Cutoff radius [m]
r_0	Equilibrium distance [m]
$\mathbf{r_i}$	Position of the ith bead [m]
r_{ij}	Distance between the ith and j^{th} beads [m]
$\hat{\mathbf{r}}_{ij}$	Unit vector along the direction from the bead position $\mathbf{r_i}$ to $\mathbf{r_j}$ [m]
R	Constant of perfect gases [8.314 $J.mol^{-1}.K^{-1}$]; Radius of SiNPs [m]
S	Surface area [m^2]
$\bar{S}$	Specific surface area ($m^2.g^{-1}$)
t	Time [s]
T	Temperature [K]
$\mathbf{v_i}$	Velocity of the ith bead [$m.s^{-1}$]
$\mathbf{v_{ij}}$	Relative velocity between the ith and j^{th} beads [$m.s^{-1}$]
V	Volume [m^3]
V_{ij}	Partial molar volume [$m^3.mol^{-1}$]
wt%	Weight loading
X	Mass fraction (-)

Greek symbols

χ_{ij}	Flory-Huggins binary interaction parameter [-]
δ	Hildebrandt / Hansen solubility parameter [$Pa^{1/2}$]
Ψ	Interfacial coverage
Φ	Interfacial coverage (with correction of contact angle)
γ	Interfacial or surface tension [$N.m^{-1}$]
Γ	Surface density [m^{-2}]
η	Friction coefficient [$J.s.m^{-2}$]
Π	Interfacial pressure [$N.m^{-1}$]

θ	Interfacial contact angle [°]
ρ	Density [beads.m^{-3}; kg.m^{-3}]
σ	Amplitude of the noise [J.m^{-1}.s$^{-1/2}$]
τ	Time unit [s]; Line tension [N]
$\omega(r_{ij})$	Weight functions [-]
ζ_{ij}	Random number between 0 and 1
Ξ	Ratio between the effective and specific surface areas of the particles

Subscripts

d	Dissipative
eff	Effective
hb	Hydrogen bonding
int	Interfacial
p	Polar; particle
SiO_2	Silica
T	Total
W	Water

Superscripts

C	Conservative
D	Dispersive
R	Repulsive

Acronyms

CMF	Critical mass fraction
DPD	Dissipative Particle Dynamics
HLB	Hydrophilic-Lipophilic Balance
MD	Molecular Dynamics
PIC	Pickering Interfacial Catalysis
SiNP	Silica nanoparticle
SiOH	Silanol group

REFERENCES

([1]) Groot, R. D.; Warren, P. B. Dissipative particle dynamics: Bridging the Gap Between Atomistic and Mesoscopic Simulation. *J. Chem. Phys.* **1997**, *107*, 4423-4435.

([2]) Español, P. Dissipative Particle Dynamics Revisited. SIMU, Challenges in Molecular Simulations. **2002**, 4, 59-77.

([3]) Shi, K.; Lian, C.; Bai, Z.; Zhao, S-L.; Liu, H. Dissipative Particle Dynamics Study of the Water/Benzene/Caprolactam System in the Absence or Presence of Non-Ionic Surfactants. *Chem, Eng. Sci*. **2015**, *122*, 185-196.

([4]) Maiti, A.; McGrother, S. Bead-Bead Interaction Parameters in Dissipative Particle Dynamics: Relation to Bead-Size, Solubility Parameter, and Surface Tension. *J. Chem. Phys.* **2004**, *120*, 1594-1601.

([5]) Lin, S.; Xu, M.; Yang, Z. Dissipative Particle Dynamics Study on the Mesostructures of n-Octadecane/Water Emulsion with Alternating Styrene-Maleic Acid Copolymers as Emulsifier. *Soft Matter* **2012**, *8*, 375-384.

([6]) Groot, R. D.; Rabone, K. L. Mesoscopic Simulation of Cell Membrane Damage, Morphology Change and Rupture by Nonionic Surfactants. *Biophys. J.* **2001**, *81*, 725-736.

([7]) Lindvig, T.; Michelsen, M. L.; Kontogeorgis, G. M. A Flory-Huggins Model Based on the Hansen Solubility Parameters. *Fluid Phase Equilib.* **2002**, *203*, 247-260.

([8]) Hansen, C. M. Hansen Solubility Parameters: A User's Handbook 2007, CRC Press, Boca Raton, FL p. 519

([9]) https://www.stevenabbott.co.uk/practical-solubility/hsp-basics.php

([10]) Thompson, A. P.; Aktulga, H. M; Berger, R.; Bolintineanu, D. S.; Brown, W. M.; Crozier, P. S.; in 't Veld, P. J.; Kohlmeyer, A.; Moore, S. G.; Nguyen, T. D.; Shan, R.; Stevens, M. J.; Tranchida, J.; Trott, C.; Plimpton, S. J. LAMMPS – a Flexible Simulation Tool for Particle-Based Materials Modeling at the Atomic, Meso, and Continuum Scales *Comp. Phys. Comm.* **2022**, *271*, 10817.

([11]) Sun, H.; COMPASS: An ab Initio Force-Field Optimized for Condensed-Phase Applications-Overview with Details on Alkane and Benzene Compounds *J. Phys. Chem. B* **1998**, *102*, 7338-7364.

([12]) Castillo, J. M.; Klos, M.; Jacobs, K.; Horsch, M. and Hasse, H. Characterization of Alkylsilane Self-Assembled Monolayers by Molecular Simulation. *Langmuir* **2015**, *31*, 2630-2638.

([13]) Pathirannahalage, S. P. K.; Meftahi, N.; Elbourne, A.; Weiss, A. C. G.; McConville, C. F.; Padua, A.; Winkler, D. A.; Gomes, M. C.; Greaves, T. L.; Le, T. C.; Besford, Q. A.; Christofferson, A. J. Systematic Comparison of the Structural and Dynamic Properties of Commonly used Water Models for Molecular Dynamics Simulations. *J. Chem. Inf. Model.* **2021**, *61*, 4521-4536.

([14]) Frisch, M. J.; Trucks, G. W.; Schlegel, H. B.; Scuseria, G. E.; Robb, M. A.; Cheeseman, J. R.; Scalmani, G.; Barone, V.; Petersson, G. A.; Nakatsuji, H.; Li, X.; Caricato, M.; Marenich, A. V.; Bloino, J.; Janesko, B. G.; Gomperts, R.; Mennucci, B.; Hratchian, H. P.; Ortiz, J. V.; Izmaylov, A. F.; Sonnenberg, J. L.; Williams Young, D., F.; Lipparini, F.; Egidi, F.; Goings, J.; Peng, B.; Petrone, A.; Henderson, T.; Ranasinghe, D.; Zakrzewski, V. G.; Gao, J.; Rega, N.; Zheng, G.; Liang, W.; Hada, M.; Ehara, M.; Toyota, K.; Fukuda, R.; Hasegawa, J.;

Ishida, M.; Nakajima, T.; Honda, Y.; Kitao, O.; Nakai, H.; Vreven, T.; Throssell, K.; Montgomery Jr, J. A.; Peralta, J. E.; Ogliaro, F.; Bearpark, M. J.; Heyd, J. J.; Brothers, E. N.; Kudin, K. N.; Staroverov,V. N.; Keith, T. A.; Kobayashi, R.; J., Normand, Raghavachari, K.; Rendell, A. P.; Burant, J. C.; Iyengar, S. S.; Tomasi, J.; Cossi, M.; Millam, J. M.; Klene, M.; Adamo, C.; Cammi, R.; Ochterski, J. W.; Martin, R. L.; Morokuma, K.; Farkas, O.; Foresman, J. B.; Fox, D. J. Gaussian 16, Rev. A.03; Gaussian, Inc.: **2016**.

([15]) Parr, R. G.; Yang, W. In *Density Functional Theory of Atoms and Molecules*; Oxford University Press: **1989**, pp. XX.

([16]) Lee, C.; Yang, W.; Parr, R. G. Development of the Colle-Salvetti Correlation-Energy Formula into a Functional of the Electron Density. *Phys. Rev. B: Condens. Matter Mater. Phys.* **1988**, *37*, 785-789.

([17]) Becke, A. D. Density-Functional Thermochemistry. III. The Role of Exact Exchange. *J. Chem. Phys.* **1993**, *98*, 5648-5652.

([18]) Stephens, P. J.; Devlin, F. J.; Chabolowski, C. F.; Frisch, M. J.Ab Initio Calculation of Vibrational Absorption and Circular Dichroism Spectra Using Density Functional Force Fields. *J. Phys. Chem.* **1994**, *98*, 11623-11627.

([19]) Hehre, W. J.; Ditchfield, R.; Pople, J. A. Self-Consistent Molecular Orbital Methods. XII. Further Extensions of Gaussian-Type Basis Sets for Use in Molecular Orbital Studies of Organic Molecules. *J. Chem. Phys.* **1972**, *56*, 2257-2261.

([20]) Hariharan, P. C.; Pople, J. A. The Influence of Polarization Functions on Molecular Orbital Hydrogenation Energies. *Theor. Chim. Acta* **1973**, *28*, 213-222.

([21]) Francl, M. M.; Pietro, W. J.; Hehre, W. J.; Binkley, J. S.; Gordon, M. S.; DeFrees, D. J.; Pople, J. A. Self-Consistent Molecular Orbital Methods. XXIII. A Polarization-Type Basis Set for Second-Row Elements. *J. Chem. Phys.* **1982**, *77*, 3654-3665.

([22]) Grimme, S.; Antony, J.; Ehrlich, S.; Krieg, H. A Consistent and Accurate Ab Initio Parametrization of Density Functional Dispersion Correction (DFT-D) for the 94 Elements H-Pu. *J. Chem. Phys.* **2010**, *132*, 154104.

([23]) Singh, J. K.; Kofke, D. A. and Errington, J. R. Surface Tension and Vapor-Liquid Phase Coexistence of Square-Well Fluid. *J. Chem. Phys.* **2003**, *119*, 3405.

([24]) Zhang, Y.; Feller, S. E.; Brooks, B. R. and Pastor, R. W. Computer Simulation of Liquid/Liquid Interfaces. I. Theory and Application to Octane/Water. *J. Chem. Phys.* **1995**, *103*, 10252-10266.

([25]) Kraft, D. J.; de Folter, J. W. J.; Luigjes, B.; Castillo, S. I. R.; Sacanna, S.; Philipse, A. P.; Kegel, W. K. Conditions for Equilibrium Solid-Stabilized Emulsions. *J. Phys. Chem. B* **2010**, *114*, 10347-10356.

([26]) Arditty, S.; Schmitt, V.; Lequeux, F.; Leal-Calderon, F. Interfacial Properties in Solid-Stabilized Emulsions. *Eur. Phys. J. B* (Condensed Matter and Complex Systems) **2005**, *44*, 381-393.

([27]) Arditty, S.; Whitby, C. P.; Binks, B. P.; Schmitt, V.; Leal-Calderon, F. Some General Features of Limited Coalescence in Solid-Stabilized Emulsions. *Eur Phys J E* (Soft Matter) **2003**, *11*, 273-281.

([28]) Binks, B. P.; Lumsdon, S. O. Pickering Emulsions Stabilized by Monodisperse Latex Particles: Effects of Particle Size. *Langmuir* **2001**, *17*, 4540-4547.

([29]) Dedovets, D.; Li, Q.; Leclercq, L.; Nardello-Rataj, V.; Leng, J.; Zhao, S-L.; Pera-Titus, M. Multiphase Microreactors Based on L-L and G-L Dispersions Stabilized by Colloidal Catalytic Particles. *Angew, Chem. Int. Ed*. **2022**, *61*, e202107537.
